# Role of sodium salicylate in *Staphylococcus aureus* quorum sensing, virulence, biofilm formation and antimicrobial susceptibility

**DOI:** 10.3389/fmicb.2022.931839

**Published:** 2022-08-01

**Authors:** Adam Benedict Turner, Erik Gerner, Rininta Firdaus, Maite Echeverz, Maria Werthén, Peter Thomsen, Sofia Almqvist, Margarita Trobos

**Affiliations:** ^1^Department of Biomaterials, University of Gothenburg, The Sahlgrenska Academy, Gothenburg, Sweden; ^2^Center for Antibiotic Resistance Research (CARe), University of Gothenburg, Gothenburg, Sweden; ^3^Mölnlycke Health Care AB, Gothenburg, Sweden; ^4^Microbial Pathogenesis Research Unit, Public University of Navarre, Pamplona, Spain

**Keywords:** *Staphylococcus aureus*, quorum sensing, biofilm, sodium salicylate, titanium, collagen, periprosthetic joint infection, wound infection

## Abstract

The widespread threat of antibiotic resistance requires new treatment options. Disrupting bacterial communication, quorum sensing (QS), has the potential to reduce pathogenesis by decreasing bacterial virulence. The aim of this study was to investigate the influence of sodium salicylate (NaSa) on *Staphylococcus aureus* QS, virulence production and biofilm formation. In *S. aureus* ATCC 25923 (*agr* III), with or without serum, NaSa (10 mM) downregulated the *agr* QS system and decreased the secretion levels of alpha-hemolysin, staphopain A and delta-hemolysin. Inhibition of *agr* expression caused a downregulation of delta-hemolysin, decreasing biofilm dispersal and increasing biofilm formation on polystyrene and titanium under static conditions. In contrast, NaSa did not increase biofilm biomass under flow but caused one log_10_ reduction in biofilm viability on polystyrene pegs, resulting in biofilms being twice as susceptible to rifampicin. A concentration-dependent effect of NaSa was further observed, where high concentrations (10 mM) decreased *agr* expression, while low concentrations (≤0.1 mM) increased *agr* expression. In *S. aureus* 8325-4 (*agr* I), a high concentration of NaSa (10 mM) decreased *hla* expression, and a low concentration of NaSa (≤1 mM) increased *rnaIII* and *hla* expression. The activity of NaSa on biofilm formation was dependent on *agr* type and material surface. Eight clinical strains isolated from prosthetic joint infection (PJI) or wound infection belonging to each of the four *agr* types were evaluated. The four PJI *S. aureus* strains did not change their biofilm phenotype with NaSa on the clinically relevant titanium surface. Half of the wound strains (*agr* III and IV) did not change the biofilm phenotype in the 3D collagen wound model. In addition, compared to the control, ATCC 25923 biofilms formed with 10 mM NaSa in the collagen model were more susceptible to silver. It is concluded that NaSa can inhibit QS in *S. aureus*, decreasing the levels of toxin production with certain modulation of biofilm formation. The effect on biofilm formation was dependent on the strain and material surface. It is suggested that the observed NaSa inhibition of bacterial communication is a potential alternative or adjuvant to traditional antibiotics.

## Introduction

*Staphylococcus aureus* is a Gram-positive commensal bacterium of the human host and occasional opportunistic pathogen (Wang and Muir, 2016). As a pathogen, *S. aureus* has the ability to cause both acute and chronic infection due to its array of virulence factors (Lowy, 1998). It causes infectious diseases such as bacteremia, sepsis, surgical site infection, and biofilm-associated infection of implanted devices and chronic wounds (Lowy, 1998; Serra et al., 2015). The various virulence factors involved in causing these infections undergo highly controlled temporal regulation depending on the growth phase in which the bacteria exist (Kong et al., 2006). The early log phase is characterized by the production of cell-surface factors that initiate, promote, and sustain attachment, adhesion, and colonization, allowing the bacterium to escape host defenses and establish biofilms (Kong et al., 2006). In the post-exponential and stationary phases, the production of secreted virulence factors is predominant, while at the same time, a downregulation of cell-surface factors prevails (Dinges et al., 2000). This change in behavior can lead to dispersal of the established biofilm and the spread of infection (Boles and Horswill, 2008; Kolodkin-Gal et al., 2010; Peschel and Otto, 2013).

The behavioral phasic changes, as well as the controlled expression of certain related virulence factors, are under the regulation of the cell density-dependent communication process known as quorum sensing (QS) (Tan et al., 2018). In *S. aureus*, one of these communication systems is controlled by the operon *accessory gene regulator (agr)*. The *agr* QS machinery regulates over 70 genes in *S. aureus*, of which more than 20 are directly involved in virulence (George and Muir, 2007). The *agr* operon is a five-gene locus responsible for the diversity of behaviors exhibited by *S. aureus*, and it contributes to various aspects of virulence. The control is mediated through the action of two separate transcripts, RNAII and RNAIII, promoted by divergent promoters P2 and P3, respectively. RNAII is responsible for the production of the QS machinery (*agrB*, *agrD*, *agrC*, and *ag*r*A*), while RNAIII is the *agr* effector molecule (Novick et al., 1993). Following transcription from the P2 promoter, the QS machinery is translated. AgrA and AgrC comprise a two-component regulatory system (TCS) responding to an autoinducing peptide (AgrD) that has undergone modification and export from the cell by AgrB. Once the concentration of this processed autoinducing peptide (AIP) achieves a certain threshold, AgrA transmits the QS signal intracellularly through a phosphorelay system (Novick et al., 1995; Lina et al., 1998; Luong et al., 2002). As the intracellular effector of *agr*, RNAIII is in control of the various *agr* target genes (Novick et al., 1993). In its action, RNAIII is bifunctional (Boisset et al., 2007), typically blocking translation and inhibiting the production of surface proteins such as staphylococcal protein A (Spa) while promoting the production of exotoxins such as alpha-hemolysin (Hla) and phenol-soluble modulins (PSM), such as delta-hemolysin (Hld), for which RNAIII acts as mRNA (Morfeldt et al., 1995; Boisset et al., 2007).

The expression of *agr* is also implicated in the regulation of *S. aureus* biofilms (Boles and Horswill, 2008), defined as a community of associated bacteria embedded in a protective extracellular polymeric matrix consisting of protein, eDNA and polysaccharides (Donlan Rodney and Costerton, 2002). Bacteria in biofilms possess modified growth kinetics, gene expression, and protein production compared to their planktonic counterparts that allow them to establish persistence; therefore, biofilm formation is an important adaptation in the development of chronic infection (Donlan Rodney and Costerton, 2002). During early infection, *agr* is highly expressed, promoting invasion and evasion from host defenses. Over time, this expression potentially decreases to allow persistence limiting the activation of the host immune response (Tuchscherr and Löffler, 2016). However, the exact role of *agr* in biofilms is known to vary depending on the strain, growth conditions and infection model under study. Under certain conditions, disruption of *agr* has no effect on biofilm formation, while in others, expression of *agr* can increase or decrease biofilm formation (Yarwood et al., 2004). For example, *agr* expression has been observed to aid the establishment of infection in a rabbit endocarditis model but prevents infection in a foreign body model (Cheung et al., 1994; Vuong et al., 2000).

The regulation and expression of virulence factors is central to pathogenesis during clinical *S. aureus* infection (Traber et al., 2008; Roy et al., 2020). By controlling the expression of virulence factors, modifying the mode of growth, and regulating bacterial metabolism (Queck et al., 2008), the *agr* QS system plays an influential role in the establishment, maintenance, and propagation of infection and in the outcome of the patient’s infection (Schweizer et al., 2011; Lee et al., 2020, 2021). As such, the possibility of modulating *agr* expression and the associated QS behavior proposes a potential method of infection control. The continuous and widespread use of traditional antimicrobial agents has resulted in an ideal environment for the promotion of resistance selection. As a result, the development of novel therapeutic approaches that present reduced or no selection pressure while still possessing anti-virulent or anti-infection properties, such as QS inhibitors, represent an interesting alternative. Research on QS inhibition has considerably increased over the last two decades as knowledge and understanding of the QS machinery in *S. aureus* has improved. Many studies have investigated potential natural (Gov et al., 2001) and/or synthetic therapeutic options to modulate *agr* (Sully et al., 2014; Mahdally et al., 2021).

Recent investigations have shown that salicylic acid has *agr* inhibitory effects, decreasing key virulence factors in *S. aureus*, such as the expression of the alpha hemolysin (*hla*) and fibronectin-binding protein A gene (*fnbA*) (Kupferwasser et al., 2003). However, previous studies also showed that salicylic acid treatment could contribute to the infection persistence of *S. aureus* in the host by different mechanisms: (i) increasing *S. aureus* invasion of epithelial cells due to reduced production of capsular polysaccharides exposing surface adhesins (Alvarez et al., 2010) and increased expression of extracellular adhesin protein (Eap) (Alvarez et al., 2011), (ii) increasing colonization and biofilm formation by inducing polysaccharide intercellular adhesin (PIA) expression due to a decrease in iron bioavailability (Dotto et al., 2017), and (iii) stabilizing mature biofilms by impairing dispersal through *agr* interference, resulting in decreased production of proteases, PSMs, and bacterial cell autolysis (Dotto et al., 2021). There is a need to further ascertain if the benefits of QS modulation in decreasing toxic factors in *S. aureus* can outweigh the potential effects in persistence of infection, especially using clinically relevant *in vitro* biofilm models. Our group has previously investigated the effect of sodium salicylate (NaSa), which is the sodium salt of salicylic acid and is several hundred-fold more soluble in water than salicylic acid, showing decreased virulence (Gerner et al., 2020) and increased susceptibility to silver in the Gram-negative pathogen *P. aeruginosa* (Gerner et al., 2021).

This investigation aimed to determine if and how NaSa modulates *agr* expression and affects the production of selected virulence factors, biofilm formation and antimicrobial susceptibility in *S. aureus*. We further aimed to assess its effect on these factors in clinically relevant *in vitro* conditions using clinical strains from periprosthetic-joint infection and chronic wound infection. Lastly, the influence of serum proteins, included in the test media at a concentration similar to that of wound fluid (Trengove et al., 1996) to simulate *in vivo* conditions, on the efficacy of NaSa was investigated in terms of viability, growth and gene expression.

## Materials and methods

### Construction of the *Staphylococcus aureus* pCN47(TT):P2(*agr*)-GFP reporter strain

General DNA manipulations were performed using standard procedures. Plasmids were purified using the NucleoSpin Plasmid miniprep kit (Macherey-Nagel, Düren, Germany) according to the manufacturer’s protocol. FastDigest restriction enzymes and Rapid DNA ligation kit (Thermo Fisher Scientific, Waltham, United States) were used according to the manufacturer’s instructions. Plasmids were transformed into the *E. coli* IM01B strain (Monk et al., 2015) and *S. aureus* by electroporation using previously described protocols (Cucarella et al., 2001). Staphylococcal electrocompetent cells were generated as previously described (Schenk and Laddaga, 1992). To generate a transcriptional fusion of the *agrBDCA* P2 promoter with the *gfpmut2* gene, the intergenic region between the *agrB* and *rnaIII* genes (260 bp) from the *S. aureus* ATCC 25923 strain was fused by overlap PCR with the *gfpmut2* gene (717 bp) amplified from pCN52 (Charpentier et al., 2004). The primers used were *Bam*HI_P2_Fw GGGGATCCCAACTATTTTCCATCACATCTCT and P2_Rv TGAAAAGTTCTTCTCCTTTACTCATTTTTACACCACTC TCCTCAC to amplify the P2 promoter and GFP_ P2_Fw GTGAGGAGAGTGGTGTAAAAATGAGTAAAGGAGAAGA ACTTTTCA and *Bam*HI_GFP_Rv CCTTATTTGTATAGTTC ATCCATGCCA to amplify GFP. The overlapping PCR product was cloned into the pJET 1.2 vector (Thermo Fisher Scientific, Waltham, United States) and then subcloned into the pCN47(TT) plasmid digested with BamHI, which was previously generated. pCN47(TT) is a derivative of the pCN47 plasmid (4), in which tRNA02_TT and SerS_TT transcriptional terminators are separated by the BamHI restriction site to use this site for gene expression, avoiding putative plasmid transcript overexpression or interference. For pCN47(TT) construction, 80-mer double-stranded DNA derived from oligonucleotides tRNA02_TT_Fw GCATGCAAATGCCTATCCAAGAGGATAGGCATTTTGGA TCCAAAAAGTGGCGACAGCTTCGTCACCACTTTTTGGC GCGCC and SerS_TT_Rv GGCGCGCCAAAAAGTGGTGAC GAAGCTGTCGCCACTTTTTGGATCCAAAATGCCTATCC TCTTGGATAGGCATTTGCATGC was digested with PaeI and SgsI prior to ligation in the pCN47 vector.

### Bacterial culture conditions for inoculum preparation and collection of bacterial supernatants

All wild-type *S. aureus* strains were streaked from –80°C stocks onto 5% horse blood Columbia agar plates (Media Department, Clinical Microbiology Laboratory, Sahlgrenska University Hospital, Sweden). Reporter strains (*S. aureus* ATCC 25923 + pCN47(TT):P2(*agr*)-GFP, *S. aureus* 8325-4 *rnaIII::lacZ, hla::lacZ* or *spa::lacZ*) were streaked from –80°C stocks onto Tryptic Soy Agar (TSA) (Merck, Darmstadt, Germany) plates containing 5 or 1.5 μg/mL erythromycin, respectively. After streaking onto the respective media, wild-type and reporter strains were incubated aerobically overnight at 37°C. For inoculum preparation, isolated colonies were then inoculated into broth cultures in one of two ways. In the first procedure, one to three colonies were selected from the agar plate and inoculated into 5 mL Tryptic Soy Broth (TSB) (Scharlau, Barcelona, Spain) and incubated at 37°C under shaking at 200 rpm for 18 h. After which, 1:100 dilutions were made into the different treatment groups for investigation (*rnaIII::lacZ, hla::lacZ* or *spa::lacZ* expression, Western blot, ddPCR). In the second procedure, colonies were selected from the agar plate and added to 4 mL TSB to achieve an OD_546_ of 0.13 (equivalent to 10^8^ CFU/mL) using a colorimeter. These suspensions were further diluted to establish inoculums of specific concentrations of the different treatment groups for several experiments [P2(*agr*)-GFP expression in plate reader, crystal violet, confocal microscopy, antimicrobial susceptibility testing].

In some experiments, *S. aureus* supernatants were used as control as a source of auto-inducing peptide (AIP), these were produced as follows. *S. aureus* ATCC 25923, periprosthetic joint infection (PJI) and wound strains were streaked from –80°C stocks onto 5% blood agar plates and incubated aerobically overnight at 37°C. Three colonies from these plates were inoculated into 15 mL TSB and incubated at 37°C shaking at 200 rpm for 24 h. After which, cultures were centrifuged at 4,500 rpm for 10 min and the supernatants were collected and 0.2 μm sterile filtered before freezing at –20°C for long-term storage.

### RNA extraction, purification and reverse transcription

From inoculums prepared following the first procedure mentioned above, a 1:100 dilution of *S. aureus* ATCC 25923 or *S. aureus* 8325-4 wild-type grown overnight was made into 5 mL TSB containing 0.01, 0.1, 1 or 10 mM NaSa (Sigma–Aldrich, Darmstadt, Germany). Three independent trials were performed with duplicate samples. Additional control groups were included: TSB (media control), TSB with 20 μM AIP-I (*agr* inhibitor control for ATCC 25923 or *agr* activator control for 8325-4) (Jenul et al., 2019) (Bachem, Bubendorf, Switzerland), TSB with 10% AIP-III supernatant (*agr* activator control for ATCC 25923 or *agr* inhibitor control for 8325-4) and TSB with 0.4% acetonitrile (ACN, diluent control for AIP-I).

After 24 h of culture at 37°C and 200 rpm, samples were divided for colony forming unit (CFU) counting or RNA extraction. For CFU counting, 100 μL was collected, vortexed and 10-fold serially diluted in 0.9% saline supplemented with 0.1% Triton X before plating on 5% blood agar plates for CFU enumeration. RNA was extracted from the bacterial cells using the mechanical and cold phenol–chloroform method as previously described (Juhlin et al., 2017). Bacterial cells were diluted 2:1 in RNAprotect (Qiagen, Hilden, Germany) for 5 min at 4°C. This was then centrifuged at 3,000 × g for 10 min. One mL of TRIzol™ and 100 μL of glass beads were added prior to homogenization with a TissueLyser II (Qiagen, Hilden, Germany) for 5 min at 30 Hz. Additional homogenization for 1 min at 18 Hz was performed after adding 200 μL of chloroform to the lysates. The aqueous phase from the phase separation was mixed with an equal volume of 70% ethanol to precipitate RNA before on-column purification. The extracted RNA was purified using the RNeasy Mini kit (Qiagen, Hilden, Germany) and eluted in 30 μL of water. The remaining DNA was removed using an RNase-free DNase set (Qiagen, Hilden, Germany) by adding 10 μL of Buffer RDD, 2.5 μL of DNase I stock solution, and 57.5 μL of RNase-free water to the RNA solution. The mixture was incubated at RT for 10 min prior to clean up using the RNeasy MinElute Cleanup Kit (Qiagen, Hilden, Germany). The concentrated RNA was stored at –80°C. RNA quality was evaluated using a Bioanalyzer 2100 (RNA 6000 Pico; Agilent Technologies, Santa Clara United States), where RNA integrity numbers (RIN) higher than five were considered acceptable (Fleige and Pfaffl, 2006). RNA quantity was assessed using RNA Quantification Assays and a fluorometer (DeNovix^®^, Wilmington, United States).

A total of 1 ng of purified RNA was converted to cDNA using a QuantiTect Reverse Transcription Kit (Qiagen, Hilden, Germany) with random primers in 20 μL reactions. No reverse transcriptase (NRT) controls were used to confirm that the gene expression was RNA-specific. The quantity of single-stranded cDNA was assessed using a Qubit™ ssDNA kit (Thermo Fisher Scientific, Waltham, United States) and a fluorometer.

Genomic DNA (gDNA) of *S. aureus* was used as a positive control in the ddPCR assays. A colony of *S. aureus* ATCC 25923 was added to 5 mL of TSB_GLU_ and incubated at 37°C and 200 rpm overnight. gDNA was extracted from the bacterial suspension using GenElute™ Bacterial Genomic DNA Kits (Sigma–Aldrich, Darmstadt, Germany) according to the manufacturer’s instructions for Gram-positive bacteria and *Staphylococcus* species. The viable cells of the bacterial suspension were quantified by CFU counting as described previously (Zaborowska et al., 2015; Juhlin et al., 2017).

### ddPCR assay

The copy number of four biofilm related genes (*agrA*, *hld*, *spa*, *icaA*) and reference gene *gyrA* in *S. aureus* ATCC 25953 and 8325-4 wild-type were quantified using the automated QX200™ Droplet Digital™ PCR system (Bio–Rad Laboratories, Hercules, United States). ddPCR mixtures were composed of 11 μL of 2 × ddPCR Supermix for Probes (No dUTP), 0.5 μL of 10 μM forward and reverse primers (Table 1), 8 μL of DNase/RNase free Milli-Q water, and 2 μL DNA sample to a final volume of 22 μL. Approximately 20,000 nanoliter-sized droplets were generated from the mixture using an automated droplet generator (QX200™ AutoDG ddPCR system (Bio–Rad Laboratories, Hercules, United States). The plate containing the resultant droplets was heat-sealed with a pierceable aluminum foil using a PX1 PCR plate sealer (Bio–Rad Laboratories, Hercules, United States) at 180°C for 5 s before being loaded into a C1000 Touch™ Thermal Cycler (Bio–Rad Laboratories, Hercules, United States) for amplification. The thermal cycling protocol was a 95°C enzyme activation step for 10 min, followed by 40 cycles of a two-step cycling protocol (95°C for 30 s and 60°C for 1 min), 4°C signal stabilization for 5 min, 90°C enzyme deactivation for 5 min, and a 4°C final hold step for an infinite time. The ramp rate between these steps was slowed to 2°C/s. Following amplification, plates containing amplified droplets were placed in a QX200™ Droplet Reader (Bio–Rad Laboratories, Hercules, United States) and assessed using QuantaSoft™ software (Bio–Rad Laboratories, Hercules, United States).

**TABLE 1:** Primers used for the ddPCR assay.

Gene	Function	Forward (5′–3′)	Reverse (3′–5′)
*gyrA*	DNA gyrase subunit A (reference gene)	CATTGCCAGATGTTCGTGAC	CCGGTGTCATACCTTGTTCA
*agrA*	Accessory gene regulator protein A	TGAAATTCGTAAGCATGACCC	CATCGCTGCAACTTTGTAGAC
*hld*	Delta-lysin	TTTGTTCACTGTGTCGATAATCCATTT	AAGGAGTGATTTCAATGGCACAAG
*spa*	Immunoglobulin G-binding protein precursor	GTGTAGGTATTGCATCTGTAACTTTAGG	GTTGAGCTTCATCGTGTTGCG
*icaA*	Intercellular adhesion protein A	GCAGTAGTTCTTGTCGCATTTCC	GTATTCCCTCTGTCTGGGCTTG

### SDS PAGE and western blot

Twenty-four hour cultures of *S. aureus* ATCC 25923, *S. aureus* ATCC 25923 + pCN47(TT):P2(*agr*)-GFP (MIC 7321; M19.436) and *S. aureus* ATCC25923 + pCN47(TT) (MIC 7636; M19.441) were prepared in TSB as described in section “RNA extraction, purification and reverse transcription.” including the same treatment and control groups. Cells were centrifuged at 4,500 rpm for 10 min and lysed using acid-washed glass beads (≤106 μm) (Sigma–Aldrich, St Louis, United States) in a TissueLyser II (Qiagen, Hilden, Germany). Protein lysates were dissolved in Laemmli or Tricine sample buffer (Bio–Rad Laboratories, Hercules, United States) and heated at 100°C for 5 min. A total of 10 μg of protein was loaded per well onto 10% TGX (Bio–Rad) or Tris-Tricine Mini-Protean precast gels (Bio–Rad) alongside Precision Plus Protein™ WesternC™ Blotting Standards (Bio–Rad Laboratories, Hercules, United States). The running time of SDS–PAGE was 1 h at 130 V. Following this, gels were transferred onto nitrocellulose membranes (Bio–Rad Laboratories, Hercules, United States) on a semi-dry transfer system. The membranes were blocked for 1.5 h with 5% non-fat milk powder (Bio–Rad Laboratories, Hercules, United States) in Tris-buffered saline-Tween (TBS-T) at RT. The membranes were incubated at 4°C overnight with the following primary antibodies: mouse anti-GFP Living Colors^®^ A.v. Monoclonal antibody (JL-8) (632381, Takara Bio, Shiga, Japan, 1:5,000), rabbit polyclonal anti-staphylococcal alpha toxin (anti-α-hemolysin; Hla) (S7531, Sigma–Aldrich, Darmstadt, Germany, 1:500), rabbit polyclonal anti-Staphylococcus aureus delta toxin (anti-d-hemolysin; Hld) (US Biological, Swampscott, United States, 1:500), rabbit polyclonal anti-protein A (Spa) (P3775, Sigma–Aldrich, Darmstadt, Germany, 1:1,000), and rabbit polyclonal anti-staphopain A (ScpA) (ab92983, Abcam, Cambridge, United Kingdom, 1:500). The membranes were washed 3 × 5 min in TBS-T prior to incubation with HRP-conjugated goat anti-mouse secondary antibody (31444, Invitrogen, Waltham, United States, 1:2,500) or HRP-conjugated goat anti-rabbit secondary antibody (sc-2004, Santa Cruz Biotechnology, Santa Cruz, United States, 1:10,000) for 1 h at RT. All antibodies were diluted in 5% non-fat milk powder in TBS-T. The membranes were washed 3 × 15 min in TBS-T followed by development with a Clarity™Western ECL Substrate detection kit (Bio–Rad Laboratories, Hercules, United States) for 5 min. Digital detection was performed using the ChemiDoc XRS + system with Image Lab Software (Bio–Rad Laboratories, Hercules, United States). Confirmation of the amount of protein loaded in each well was performed by stripping the blots with Restore™ Western Blot Stripping Buffer (Thermo Fisher Scientific, Waltham, United States) for 30 min at RT and reprobing with mouse anti-GAPDH (MA5-15738, Invitrogen, Waltham, United States, 1:1,000). Reactive bands in the scanned blots were quantified with ImageJ software (Supplementary Materials 1–6). The area of each band was compared to the untreated sample (relative density). Then, the relative density of each sample was compared to the relative density of the loading control (adjusted density/fold change).

### Effect of NaSa on *agr* expression in *Staphylococcus aureus* using a reporter system

Inoculums of *S. aureus* ATCC 25923 + pCN47(TT):P2(*agr*)-GFP (MIC 7321; M19.436), and the same strain but with empty plasmid *S. aureus* ATCC 25923 + pCN47(TT) (MIC 7636; M19.441) were prepared following the OD_546_ method mentioned above. These were diluted 1:10 into TSB containing 0.1, 1 or 10 mM sodium salicylate to achieve 10^7^ CFU/mL. TSB alone (media control) and 1 μM AIP-I (*agr* inhibitor control) were used as controls. Two hundred microliters of each sample in triplicate was added to clear-bottom black 96-well microtiter plates (Greiner Bio-One, Kremsmünster, Austria). Optical density at 600 nm and fluorescence intensity at 485/520 ex/em was measured every 30 min for 48 h at 37°C using a microplate reader (FluostarOmega, BMG LABTECH, Ortenberg, Germany). The fluorescence intensity of the strain with empty plasmid was used as a blank to correct for non-specific background fluorescence. The expression of *agrBDCA* genes under the P2 promoter is presented as blank-corrected fluorescence normalized by OD after 24 and 48 h of growth. Three independent trials were performed with triplicate samples.

### Role of NaSa in *Staphylococcus aureus* ATCC 25923 growth kinetics and viability

The effect of NaSa on the growth kinetics of *S. aureus* ATCC 25923 was evaluated at a cell density of 10^7^ CFU/ml in TSB, with and without 50% fetal bovine serum (HyClone Laboratories, Logan, United States), and supplemented with 0.1, 1 or 10 mM NaSa. TSB alone (media control), the inhibition controls: TSB supplemented with 10% glucose (Merck, Darmstadt, Germany), 1 μM AIP-I and 10% AIP-II containing supernatant, as well as the *agr* activator control AIP-III containing supernatant were included. Two hundred microliters of each sample in duplicate was added to 96-well microtiter plates (Thermo Fisher Scientific, Waltham, United States), and the OD at 600 nm was measured at 37°C every 30 min for 24 h using a microplate reader. After the final read, 100 μL were taken to perform CFU counting as described above.

### Effect of NaSa on biofilm formation

Cultures of *S. aureus* ATCC 25923, four *S. aureus* wound isolates and four *S. aureus* isolates from periprosthetic joint infections were prepared following the OD_546_ method described above in TSB supplemented with either NaSa (0.01 mM, 0.1 mM, 1 mM and 10 mM), TSB (media control), AIP-I (20 μM, inhibitor control), or acetonitrile (0.4%, AIP-I diluent control). One milliliter of each inoculum was added to untreated polystyrene (PS) (Thermo Fisher, Waltham, United States), collagen-coated polystyrene microtiter plates (COL) (Merck, Darmstadt, Germany) and onto titanium disc surfaces (Ti) (Christers Finmekaniska AB, Skövde, Sweden), followed by static incubation at 37°C for 24 h.

*S. aureus* 25923 was grown on all three surfaces, while the four different *S. aureus* wound isolates were grown on collagen and polystyrene surfaces and the four *S. aureus* isolates from periprosthetic joint infections were grown on titanium and polystyrene. The different *agr* types (I–IV) were represented among the isolates. After incubation, the plates were gently rinsed three times in tap water and stained for 5 min with (2%) crystal violet (VWR Chemicals BDH, Leuven, Belgium). Biofilms were then rinsed again in tap water in triplicate to remove excess dye and allowed to dry for at least 15 min. Crystal violet was solubilized in an 80:20 ethanol:acetone solution for 5 min before being transferred to a clean 96-well plate (Thermo Fisher Scientific Biolite™, Waltham, United States) and then read using a microplate reader at 595 nm. Blank wells with only media were included.

The effect of NaSa on biofilm formation was also observed using confocal laser scanning microscopy. Biofilms from ATCC 25923 were cultured with a starting inoculum of 10^5^ CFU/mL (diluted 1:1,000 from the OD_546_ method mentioned above) in 35 mm petri dishes (Thermo Fisher Scientific, Waltham, United States) in either TSB or TSB supplemented with NaSa (10 mM). These were statically incubated at 37°C for 24 h. Upon completion of incubation, biofilms were gently rinsed three times in sterile saline (0.9%) before staining with the Filmtracer™ LIVE/DEAD™ Biofilm Viability Kit (Invitrogen, Waltham, United States) at room temperature for 20 min in the dark. After staining, biofilms were again rinsed three times in sterile saline (0.9%) and imaged using a Nikon C2^+^ confocal laser-scanning microscope (Nikon, Tokyo, Japan) and a 100 × water dipping objective (CFI Plan 100XC W). Z-stacks were taken at 3 μM slices through the biofilm, and biofilm thickness and biomass were analyzed in COMSTAT 2.1 (Heydorn et al., 2000). Five z-stacks were taken at randomly chosen fields of view on each sample. Three independent trials were performed with duplicate samples.

### Effect of NaSa on biofilm dispersal

The dispersal of *S. aureus* ATCC 25923 biofilms was measured by viable colony counting of dispersed planktonic cells in the media surrounding the biofilm and in the biofilm. Biofilms were established by seeding 10^5^ CFU/mL (diluted 1:1,000 from the OD_546_ method mentioned above) in 48-well microtiter plates (Thermo Fisher Scientific, Waltham, United States) and grown statically either in TSB or NaSa (0.01 mM, 0.1 mM, 1 mM, and 10 mM) for 24 h at 37°C. Upon reaching 24 h, the planktonic phase was removed, and viable CFU counting was performed following the above-mentioned procedure. Fresh media containing the respective treatment conditions was then added to the biofilms and incubated for an additional 24 h. After completion of the secondary incubation, the new planktonic phase was removed, and viable colony counting was performed. Viable colony counting of the biofilm was also performed at this point. For the planktonic phase, CFU counting was performed as mentioned above. For the cells in the biofilm, biofilms were gently rinsed three times with sterile saline (0.9%) and then suspended in 1 mL of sterile saline (0.9%). Biofilms were sonicated in plate (42 kHz) for 30 s to release adherent cells and then resuspended by pipetting to ensure homogenization. The resuspended biofilms were then 10-fold serially diluted in saline (0.9%) and Triton-X (0.1%) and plated on horse-blood agar (5%). The plates were incubated at 37°C, and viable colonies were counted after overnight incubation. Three independent trials were performed, each with two technical replicates.

### Expression of *rnaIII*, *hla* and *spa* using reporter strains

#### Agar beta-galactosidase assay

The agar beta-galactosidase assay was modified based on a previously described protocol (Canovas et al., 2016). *S. aureus* 8325-4 *rnaIII::lacZ, hla::lacZ* or *spa::lacZ* were inoculated in 5 mL of TSB containing 5 μg/mL erythromycin and incubated at 37°C and 200 rpm for 20 h. These were diluted 1:1,000 and combined with liquid TSA supplemented with 10 μg/mL erythromycin and 150 μg/mL X-gal. This was then deposited into a sterile 90 mm petri dish and allowed to solidify. Then, 4 mm holes were punched out of the solidified agar plates using a biopsy punch, to which 50 μL of the desired reagents were added: NaSa (1, 10, 100 mM). Sterile water (negative control), glucose (20%, *agr* inhibition control), AIP-I (1 μM, *agr* activation control), AIP-I-containing supernatant (10%, *agr* activation control) and AIP-II-containing supernatant (10%, *agr* inhibition control) were used as controls. The plates were then incubated for 20 h at 37°C. Upon completion of incubation, plates were visually assessed for color change and the relative levels of inhibition of reporter genes. Three independent trials were performed, with duplicate samples.

#### Liquid beta-galactosidase assay

The effect of NaSa on *rnaIII, hla*, and *spa* expression was further evaluated using the liquid beta galactosidase activity assay (Chan and Foster, 1998). Overnight cultures of *S. aureus* 8325-4 *rnaIII::lacZ, hla::lacZ* or *spa::lacZ* (Chan and Foster, 1998) in TSB were diluted 1:100 in TSB supplemented with 5 μg/mL erythromycin and 0, 0.1, 1, or 10 mM NaSa, with or without 50% fetal bovine serum. TSB with AIP-I (1 μM, *agr* activator control), 20% glucose (*agr* inhibition control), 10% supernatant containing AIP-I (*agr* activator control) and AIP-II (*agr* inhibition control) were used as controls. The cultures were incubated for 4 and 24 h at 37°C and 200 rpm before centrifuging the cultures at 14,000 rpm (18,620 *g*) for 5 min. The pellets were washed in 1 mL PBS, resuspended in 600 μL PBS and lysed using acid-washed glass beads (≤106 μm, Sigma–Aldrich, St Louis, United States) in a TissueLyser II. After a centrifugation step at 14,000 rpm (18,620 *g*) for 5 min, 400 μL of the lysate was collected. The total protein concentration was analyzed using the BCA assay (Smith et al., 1985). Gene expression was estimated by measuring β-galactosidase activity in Miller units. One hundred microliters of lysate was mixed with 200 μL of ortho-nitrophenyl-β-galactoside (ONPG) (4 mg/mL) and incubated until yellow color development. The reaction was stopped by the addition of 500 μL of 1 M Na_2_CO_3_. OD was measured in a 96-well plate at 420 nm and used to calculate Miller units. Three independent trials were performed, each with two technical replicates.

#### *Staphylococcus aureus* biofilm susceptibility to silver sulfate and antibiotics after NaSa pretreatment

The effect of NaSa on biofilm susceptibility using silver was performed as described previously (Gerner et al., 2021), but *S. aureus* ATCC 25923 was used to establish a biofilm for 24 h without inclusion of serum. Briefly, semisolid collagen type I gels with 0, 0.1, 1 or 10 mM NaSa were inoculated with 5 μL 10^8^ CFU/mL bacteria. After 24 h of biofilm establishment, silver sulfate (Merck, Darmstadt, Germany) was added to generate 0–500 ppm Ag in the gels. After 24 h of treatment, the gels were solubilized with collagenase, and viable counts were quantified using above mentioned CFU counting. The effect of NaSa on biofilm susceptibility was also tested on titanium surfaces and on polystyrene microtiter plates. *S. aureus* ATCC 25923 biofilms treated with NaSa (0 mM, 0.1 mM, 1 mM and 10 mM) were allowed to establish statically at 37°C for 24 h, after which they were treated with varying concentrations of silver sulfate (500, 100, 50, 10, 0 ppm) and incubated again statically at 37°C for a following 24 h. After the second incubation, viable colonies were quantified following the above mentioned CFU counting.

*S. aureus* ATCC 25923 biofilms with or without NaSa (10 mM) were challenged with eight antibiotics (Supplementary Figure 7): rifampicin (0.016–1,024 μg/mL), oxacillin (2–8 μg/mL), vancomycin (1–8 μg/mL), linezolid (0.5–1,024 μg/mL), fusidic acid (0.125–1,024 μg/mL), clindamycin (0.06–1,024 μg/mL), trimethoprim-sulfamethoxazole (1–64 and 19–1,216 μg/mL) and levofloxacin (0.016–1,024 μg/mL) using the minimal biofilm eradication concentration (MBEC) method. Cultures of *S. aureus* ATCC 25923 at 5 × 10^5^CFU/mL in TSB were added to each well of a Calgary biofilm plate (Innovotech, Edmonton, Canada). The plates were then incubated for 24 h at 37°C and 125 rpm. After incubation, the biofilms were carefully rinsed by dipping the peg lid into a 96-well plate containing 0.9% saline for 1 min. At this point, three pegs were carefully removed from the plate lid and transferred to a glass tube containing 1 mL of sterile saline (0.9%) for viable colony counting. The glass tubes containing the removed pegs were sonicated (42 kHz) for 30 s, followed by vortexing at 10,000 rpm for 1 min to release all adherent biofilm bacteria. Viable colony counting was then performed on the released bacteria following the above-mentioned method.

The peg lid was transferred to a 96-well plate containing 100 μL of cation adjusted Mueller-Hinton broth (MHB) and the eight respective antibiotics and was incubated statically at 37°C for 20 h. Subsequently, the peg lids were rinsed twice in saline (0.9%) for 1 min each and transferred to a recovery plate containing cation adjusted MHB and universal neutralizer (50 mg/mL L-histidine, 50 mg/mL L-cysteine, 100 mg/mL reduced glutathione). The plates were sonicated (42 kHz) for 1 min to release the adherent biofilm cells into the recovery media. The peg lid was then removed, and the plates were covered with a new sterile lid and incubated statically overnight at 37°C. Following incubation, MBEC values for each antibiotic were obtained by visually observing the first non-turbid concentration.

#### Statistics

Statistical analyses were performed in GraphPad Prism 9.0 (GraphPad, San Diego, United States). For reporter strain gene expression (*n* = 3) using beta-galactosidase and fluorescent assays, crystal violet biomass (*n* = 3), biofilm dispersal (*n* = 3), silver susceptibility (*n* = 3), and viable colony counting (*n* = 3), statistical significance was tested using one-way ANOVA for multiple comparisons between groups. Dunnett’s *post-hoc* test was applied to evaluate differences between the media control (TSB or TSB + serum) and the different treatments. For viable biofilm colony counting from MBEC pegs (*n* = 3) and quantitative analysis of biofilm biomass and thickness (*n* = 3), statistical significance was tested using a Student’s *t*-test. *P-*values equal to or less than 0.05 were considered statistically significant (*P ≤ 0.05, ^**^P ≤ 0.01, ^***^P ≤ 0.001, ^****^P ≤ 0.0001). All statistical tests are against the TSB control, and data are expressed as the mean ± standard deviation.

## Results

### NaSa modulates quorum sensing and virulence in agr III-type strain ATCC 25923

**FIGURE 1:**
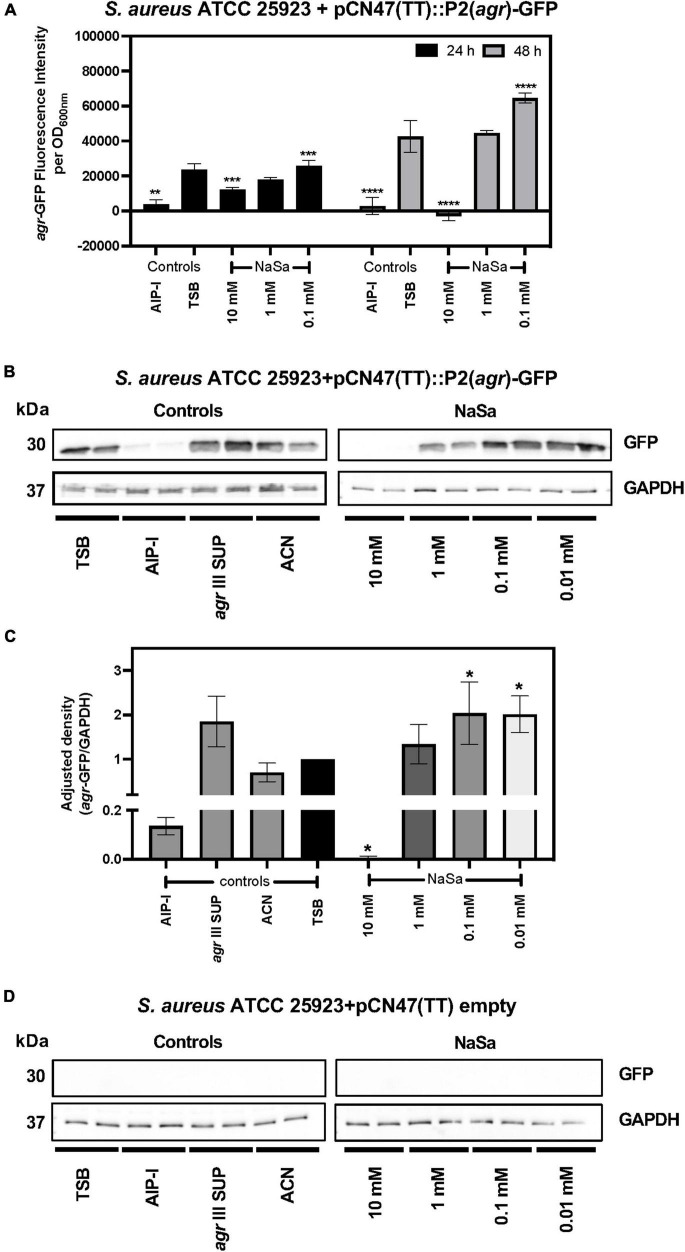
NaSa affects the expression of *agr* in *S. aureus*. *S. aureus* ATCC 25923 + pCN47(TT):P2(agr)-GFP was used as a reporter strain to evaluate the potential quorum sensing inhibitory effects of NaSa. The effect of NaSa on *agr*-GFP fluorescence intensity after 24 and 48 h is shown in **(A)**, while the 24 h effect of NaSa on GFP production as result of the expression of *agrBDCA* under the P2 promoter is shown in **(B,C)**. GAPDH was used as a loading control for semiquantitative purposes **(C–E)**. A control strain with empty plasmid was used as negative control in **(D)**. The display images **(B,D)** are representative of three independent experiments. Each bar represents the mean from three independent experiments ± *SD*. *P ≤ 0.05, **P ≤ 0.01, ***P ≤ 0.001, ****P ≤ 0.0001, statistically significant compared with TSB (Tryptic Soy Broth) as media using one-way ANOVA Dunnett’s test. AIP-I, Autoinducing Peptide-I; SUP, supernatant; ACN, acetonitrile, used as AIP-I diluent control.

**FIGURE 2:**
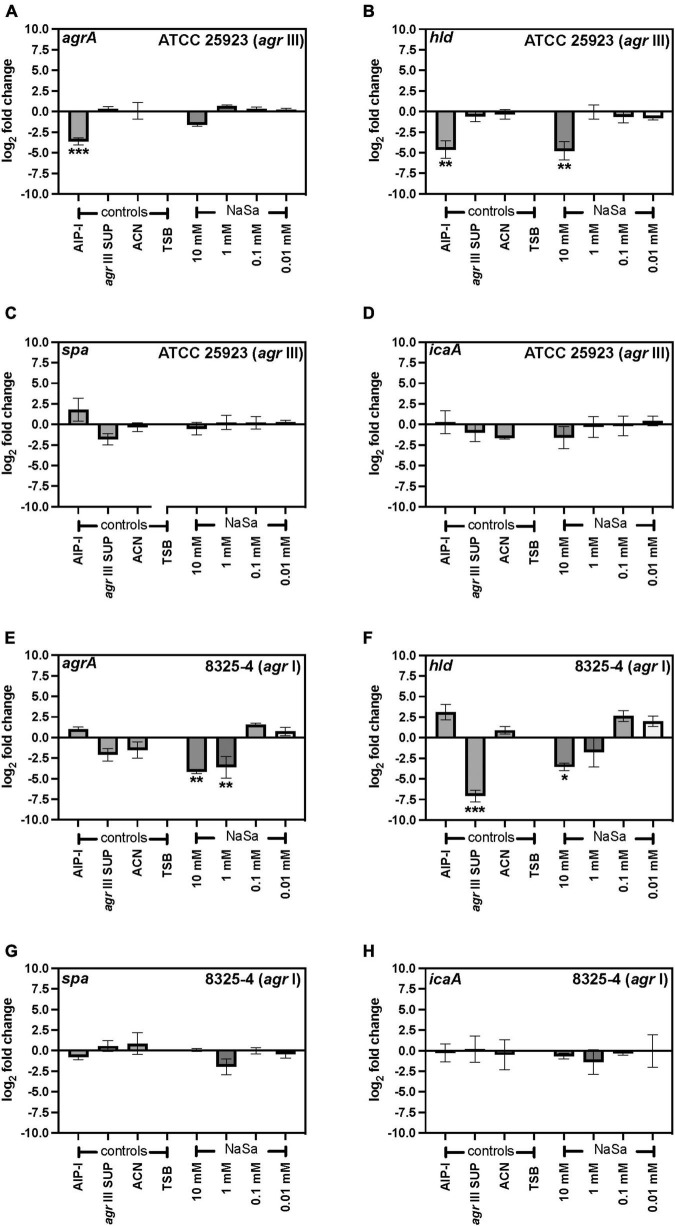
NaSa inhibits the expression of *agrA* and *hld*, but not *spa* and *icaA*, at the mRNA level. Gene expression was assessed using ddPCR assays of *agrA* **(A,E)**, *hld* **(B,F)**, *spa* **(C,G),** and *icaA* **(D,H)** in *S. aureus* ATCC 25923 **(A–D)** and 8325-4 wild-type **(E–H)**. *gyrA* was used as a reference gene. Data represent the means from three independent experiments ± *SD*. *P ≤ 0.05, **P ≤ 0.01, ***P ≤ 0.001, statistically significant to TSB (Tryptic Soy Broth) as media using one-way ANOVA Dunnett’s test. AIP-I, Autoinducing Peptide-I; SUP, supernatant; ACN, acetonitrile, used as AIP-I diluent control.

The *agr* type III *Staphylococcus aureus* ATCC 25923 strain was grown in the absence or presence of sodium salicylate (NaSa), and different phenotypic and molecular features associated with virulence were evaluated. First, a reporter strain ATCC 25923 + pCN47(TT):P2(*agr*)-GFP was created as a tool to measure the gene expression of the accessory gene regulator (*agr*) quorum sensing system. The green-fluorescence protein (GFP) was linked to the promoter P2 upstream of *agr* as confirmed by DNA sequencing of the construct, allowing the measurement of the fluorescence of GFP as an indirect determination of the expression *of agrBDCA* genes under the P2 promoter. According to plate readings (Figure 1A) and western blot (Figures 1B–D and Supplementary Materials 1, 2), the gene expression of 10 mM NaSa significantly decreased *agr* expression after 24 and 48 h of culturing (Figures 1A–C). Interestingly, low doses of NaSa (0.1 and 0.01 mM) showed a significant increase in *agr* expression at 24 h (Figure 1C) and at 48 h (Figure 1A). Additional control groups were included, such as glucose (GLU), which is known to inhibit the expression of *agr* in *S. aureus* (Regassa et al., 1991); autoinducing peptide type 1 (AIP-I), with a cross-inhibitory effect on AIP-III produced by ATCC 25923 (Malone et al., 2007); and cell-free supernatants from the same strain ATCC 25923 (*agr* III SUP), supposedly containing AIP-III (not quantified) and therefore with a potential upregulation of *agr*. Regarding the controls, as expected, AIP-I downregulated *agr* expression, while *agr* III SUP upregulated *agr* expression (Figures 1A,C). ddPCR analysis of *agrA* expression in the wild-type ATCC 25923 showed a decreasing trend with 10 mM NaSa, although the difference was not statistically significant (Figure 2A). Significantly lower expression of *hld* was detected after exposure to the highest NaSa concentration (10 mM) (Figure 2B). In contrast, irrespective of NaSa concentration, no significant changes in the expression levels of *agrA*, *spa* and *icaA* were found (Figures 2A–D). AIP-I downregulated *agrA* (Figure 2A) and *hld* expression (Figure 2B).

**FIGURE 3:**
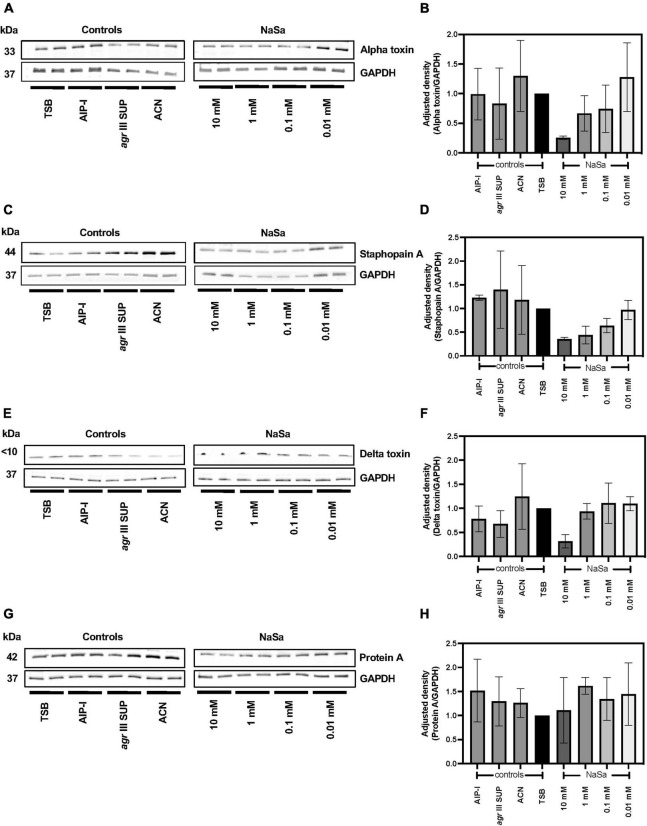
NaSa decreases production of virulence factors in *S. aureus*. The production of alpha toxin **(A,B)**, staphopain A **(C,D)**, delta toxin **(E,F)**, and protein A **(G,H)** was analyzed using western blot and densitometry analysis. GAPDH was used as a loading control. The displayed images are representative of three independent experiments. Each bar represents the mean from three independent experiments ± *SD*. 10 mM NaSa showed a decreasing trend in the secretion of alpha toxin, staphopain and delta toxin compared to TSB (Tryptic Soy Broth) as media using one-way ANOVA Dunnett’s test. AIP-I, Autoinducing Peptide-I; SUP, supernatant; ACN, acetonitrile, used as AIP-I diluent control.

We further evaluated the protein secretion of four virulence factors regulated by the *agr* QS system following NaSa treatment, including alpha hemolysin (Hla), delta hemolysin (Hld), staphopain A (ScpA) (all positively regulated) and protein A (negatively regulated). The highest concentration of NaSa (10 mM) showed a decreasing trend in the secretion of Hla (Figures 3A,B and Supplementary Material 3), ScpA (Figures 3C,D and Supplementary Material 4) and Hld (Figures 3E,F and Supplementary Material 5), whereas the secretion of Protein A (Figures 3G,H and Supplementary Material 6) was not affected by NaSa.

### NaSa modulates quorum sensing and virulence in the agr I-type strain 8325-4

**FIGURE 4:**
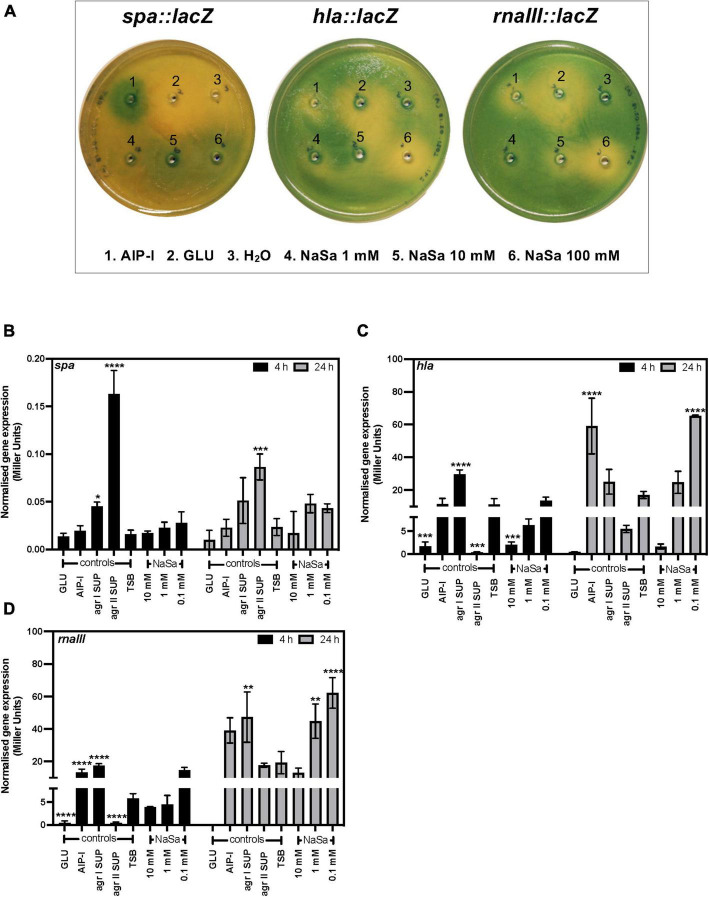
NaSa modulates the expression of *spa, hla* and *rnaIII.* Effect of NaSa on the β-galactosidase activity of *S. aureus* reporter strains *8325-4 spa::lacZ*, 8325-4 *hla*::*lacZ, and* 8325-4 *rnaIII*::*lacZ* in X-gal-containing agar plates after 24 h **(A)** or the ONPG assay after 4 and 24 h **(B–D)**. Each bar represents the mean ± *SD* from three independent experiments. *P ≤ 0.05, **P ≤ 0.01, ***P ≤ 0.001, ****P ≤ 0.0001, statistically significant to TSB (Tryptic Soy Broth) as media using one-way ANOVA Dunnett’s test. GLU, glucose; AIP-I, Autoinducing Peptide-I; SUP, supernatant; ACN, acetonitrile, used as AIP-I diluent control.

After 24 h culture, *S. aureus* 8325-4 viable CFU counts were similar between groups ranging between 9.6 and 10.6 log_10_ CFU/mL, except for 10 mM NaSa that showed a 2-log_10_ reduction in viable counts (7.5 log_10_ CFU/mL) (data not shown). In *S. aureus* 8325-4 wild-type the expression of *agrA* and *hld* (Figures 2E,F) was significantly downregulated by ≥ 1 mM NaSa, whereas NaSa treatment did not affect the expression of *spa* and *icaA* (Figures 2G,H). In addition, *S. aureus* 8325-4 reporter strains (*spa::lacZ, hla::lacZ*, and *rnaIII::lacZ*) were assessed using agar-based and liquid-based beta-galactosidase assays. With a functional *lacZ* attached to the genes of interest, the respective levels of expression could be observed because beta-galactosidase breaks down the galactose in X-gal, releasing the attached indole and resulting in a visible color change. In the agar-based assay, NaSa treatment (1 mM, 10 mM, 100 mM) resulted in a concentration-dependent inhibition of *hla* and *rnaIII* and a concentration-dependent increase in *spa*, with increasing zones of inhibition or expression observed as the NaSa concentration increased (Figure 4A). This corresponds with the bifunctional upregulation/downregulation of each of these genes by *agr* and showed proof of concept of sodium salicylate functioning as a quorum sensing inhibitor.

In the liquid-based assay, treatment with NaSa (0.1, 1, 10 mM) produced no effect on *spa* after 4 h, but an increasing trend in expression at ≤ 1 mM was observed after 24 h (Figure 4B). A reduction in the expression of *hla* was found at both 4 and 24 h after treatment with NaSa (10 mM). However, after 24 h, treatment with NaSa (0.1 mM) resulted in a significant increase in the expression of *hla* (Figure 4C). A similar trend was observed in *rnaIII* after NaSa treatment, in which a significant increase in expression was observed after 24 h (1, 0.1 mM) (Figure 4D), although in this case, no reduction in expression was observed in any treatment after either time point.

### NaSa increases biofilm formation on different material surfaces in *agr* III type ATCC 25923

**FIGURE 5:**
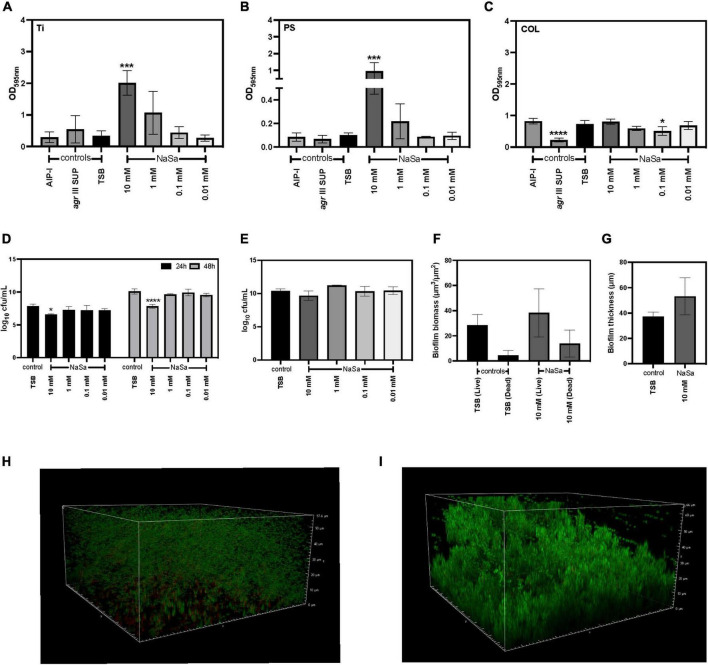
NaSa influences biofilm formation of *S. aureus* ATCC 25923. The effect of NaSa on the production of biofilm on titanium (Ti) **(A)**, polystyrene (PS) **(B)**, and collagen-coated polystyrene (COL) **(C)** was assessed after 24 and 48 h. The number of dispersed (24 and 48 h) **(D)** and surface-attached (48 h) **(E)** bacteria. Effect of NaSa on *S. aureus* biofilm biomass **(F)** and thickness **(G)** after 24 h of growth on polystyrene. Untreated *S. aureus* biofilms **(H)** and NaSa-treated *S. aureus* biofilms **(I)** grown on polystyrene for 24 h visualized by confocal laser-scanning microscopy. Each bar represents the mean ± *SD* from three independent experiments. *P ≤ 0.05, ***P ≤ 0.001, ****P ≤ 0.0001, statistically significant to TSB (Tryptic Soy Broth) as media using one-way ANOVA Dunnett’s test. AIP-I, Autoinducing Peptide-I; SUP, supernatant.

The ability of *S. aureus* ATCC 25923 to form biofilms on different material surfaces was assessed using the microtiter plate test (crystal violet). In a concentration-dependent manner, NaSa significantly increased the total biofilm biomass on titanium (10 mM) (Figure 5A) and polystyrene (10 mM) (Figure 5A) but resulted in a reduction in biofilm biomass on collagen surfaces (0.1 mM) (Figure 5C). NaSa (10 mM) significantly reduced biofilm dispersal, as observed by a decrease in CFUs released from the biofilm to the planktonic phase (Figure 5D), while CFU counts in the biofilm after 48 h were similar in all groups on polystyrene (Figure 5E). Although not significant, the biomass and maximum thickness were higher in biofilms grown with 10 mM NaSa compared to the control on polystyrene petri plates using CLSM (Figures 5F–I).

### The effect of NaSa on biofilm formation is material surface and strain dependent

The activity of NaSa on biofilm formation was dependent on strain and material surface. We assessed the *in vitro* biofilm formation of eight clinical strains from PJI and wound infection, each belonging to *agr* I, II, III and IV type, on three material surfaces [polystyrene (PS), titanium (Ti) and collagen (COL)].

**FIGURE 6:**
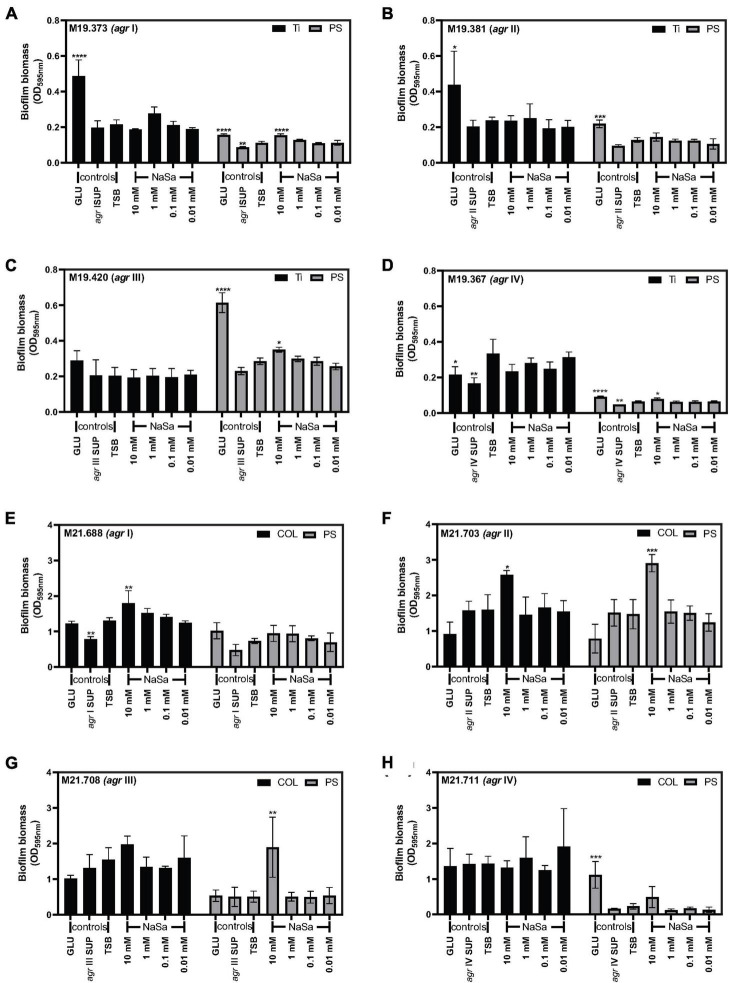
Biofilm formation influenced by NaSa depends on the strain *agr* type and material substrate. Effect of NaSa on biofilm formation after 24 h on polystyrene (PS) and titanium (Ti) by isolates from periprosthetic joint infections **(A–D)** and on polystyrene and collagen-coated polystyrene (COL) by isolates from wound infections **(E–H)**. Biofilm biomass was evaluated using crystal violet. Data represent the means from three independent experiments ± *SD*. *P ≤ 0.05, **P ≤ 0.01, ***P≤ 0.001, ****P ≤ 0.0001, statistically significant to TSB (Tryptic Soy Broth) as media using one-way ANOVA Dunnett’s test. GLU, glucose; SUP, supernatant from the respective *S. aureus* clinical strain.

Regarding the clinical isolation source and material surface, the four PJI *S. aureus* strains did not change their biofilm phenotype with NaSa on the clinically relevant titanium surface (Figures 6A–D), but 10 mM NaSa increased biofilm formation on polystyrene in *agr* I, III, IV types (Figures 6A,C,D). Half of the wound strains, except for *agr* I and II strains (Figures 6E,F), NaSa did not change the biofilm phenotype of *agr* III and IV strains on the clinically relevant collagen-coated surface (Figures 6G,H), whereas *agr* II and III biofilms on PS were promoted with 10 mM NaSa (Figures 6F,G).

When considering the *agr* type, *agr* type I strains from chronic wounds and PJI showed no change in biofilm formation after NaSa treatment on collagen and titanium (Figures 6A,E), whereas the highest (10 mM) NaSa showed a significant increase in biofilm biomass of the PJI strain on polystyrene (Figure 6A). In contrast, the *agr* II PJI strain exhibited no change in biofilm formation on PS or Ti (Figure 6B), while an increase in biofilm formation was observed under the 10 mM condition for the *agr* II wound strain on both PS and collagen (Figure 6F). The *agr* III clinical isolates showed no change in biofilm formation on Ti or COL but had a significant increase in biofilm formation with 10 mM NaSa treatment on polystyrene (Figures 6C,G), corresponding with the *agr* III ATCC 25923 lab strain (Figure 5B). NaSa did not affect biofilm formation of the *agr* IV clinical strains (Figures 6D,H), except for a minor increase in biofilm formation on PS for the PJI strain (Figure 6D).

### Pretreatment of *Staphylococcus aureus* with NaSa does not increase biofilm tolerance toward antibiotics and antiseptics

**FIGURE 7:**
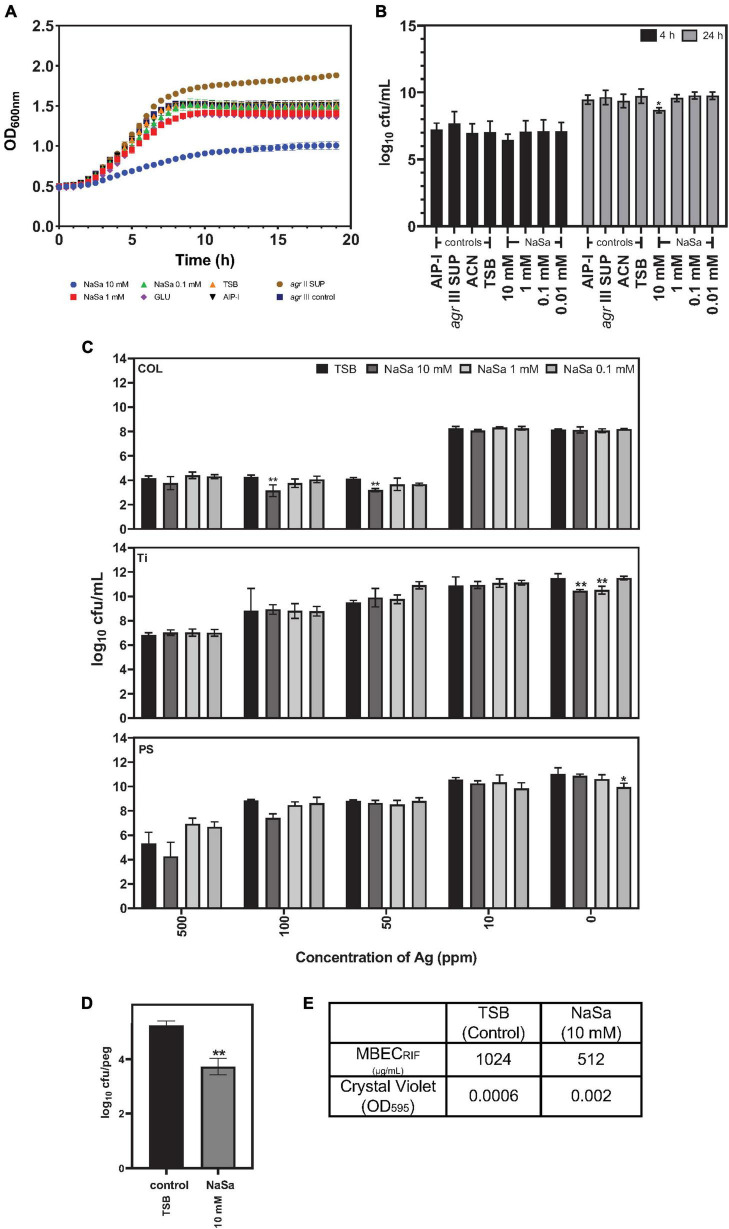
NaSa affects *S. aureus* planktonic cell viability and biofilm tolerance toward antimicrobials. Effect of NaSa on growth kinetics during 24 h **(A)** and viability after 4 h and 24 h **(B)** of planktonic *S. aureus* ATCC 25923. Influence of NaSa pretreatment on the action of silver (Ag) against *S. aureus* grown in a 3D collagen model (COL), titanium (Ti) and polystyrene (PS) **(C)**. Effect of NaSa on the formation of biofilms on pegs of the Calgary biofilm device **(D)** and its tolerance toward rifampicin (RIF) **(E)**. Data represent the means from three independent experiments ± *SD*. *P ≤ 0.05, **P ≤ 0.01, statistically significant to TSB (Tryptic Soy Broth) as media using one-way ANOVA Dunnett’s test. GLU, glucose; AIP-I, Autoinducing Peptide-I; SUP, supernatant; ACN, acetonitrile, used as AIP-I diluent control.

The influence of NaSa on *S. aureus* growth in TSB was evaluated by kinetic OD measurements in microtiter plates for 24 h followed by CFU counting. Ten millimolar NaSa decreased growth curve kinetics (Figure 7A) and 24 h CFU counts by 0.7 log_10_ units compared to control TSB (Figure 7B). Silver sulfate (Ag) and eight antimicrobial agents, commonly used in the clinical treatment of *S. aureus* infections, were used to evaluate the susceptibility of *S. aureus* biofilms pre-established in the presence or absence of NaSa. For *S. aureus* biofilms in collagen gels, 10 mM NaSa in combination with 50–100 ppm Ag resulted in one log_10_ reduction in CFU compared to same silver concentrations but without NaSa (Figure 7C). NaSa did not change the effect of silver against biofilms grown on polystyrene or titanium.

The influence of NaSa pretreatment on *S. aureus* biofilm susceptibility to antibiotics was assessed using the minimum biofilm eradication concentration (MBEC) method. The number of viable colonies in the biofilms grown on pegs was reduced by NaSa treatment (Figure 7D); however, biofilm biomass was equivalent (Figure 7E). For seven antimicrobial agents (oxacillin, vancomycin, linezolid, fusidic acid, clindamycin, trimethoprim-sulfamethoxazole, and levofloxacin), no change in MBEC was observed when grown in NaSa. However, the MBEC of rifampicin was reduced twofold from 1,024 to 512 μg/mL for biofilms grown in the presence of 10 mM NaSa compared to the untreated control (Figure 7E).

**FIGURE 8:**
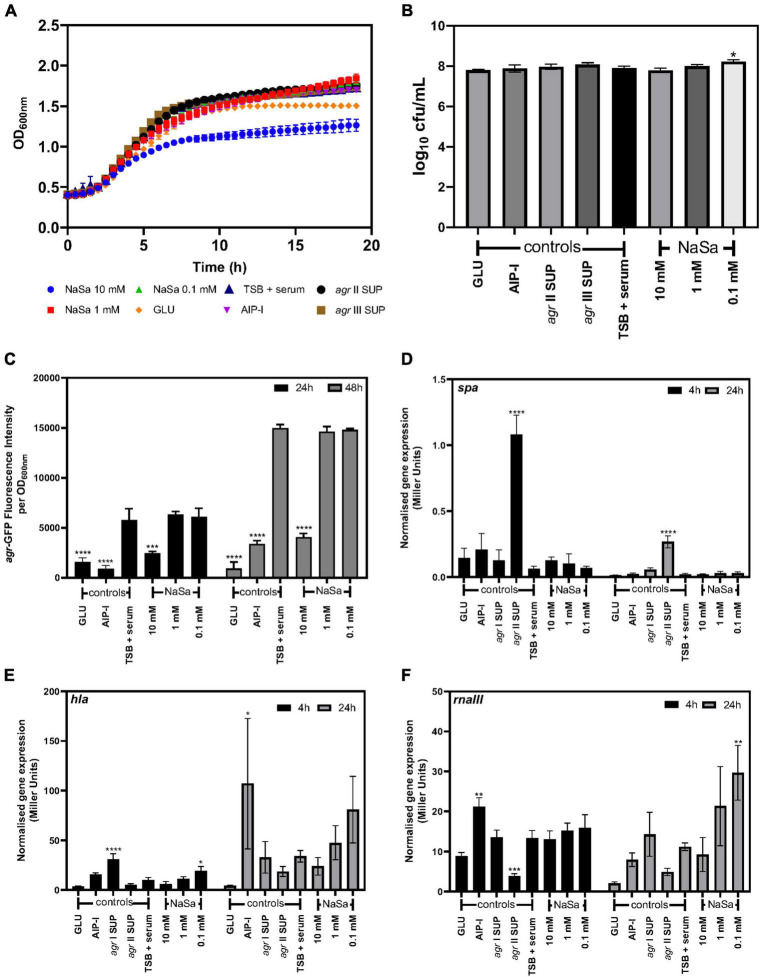
Effect of NaSa on *S. aureus agr* expression in the presence of serum (TSB + 50% serum). Growth curves **(A)** and 24 h CFU counts of *S. aureus* ATCC 25923 **(A,B)**. Influence of NaSa on *agr* expression using the *S. aureus* ATCC 25923 + pCN47(TT):P2(*agr*)-GFP reporter strain **(C)**. Effect of NaSa on the expression of *spa* **(D)**, *hla* **(E)**, and *rnaIII* **(F)** in 8325-4 *spa::lacZ*, 8325-4 *hla::lacZ*, and 8325-4 *rnaIII::lacZ* reporter strains cultured with serum (50%) for 4 and 24 h using the liquid ONPG beta galactosidase assay. Data represent the means from three independent experiments ± *SD*. *P ≤ 0.05, **P ≤ 0.01, ***P ≤ 0.001, ****P ≤ 0.0001, statistically significant to TSB (Tryptic Soy Broth) + 50% serum as media using one-way ANOVA Dunnett’s test. GLU, glucose; AIP-I, Autoinducing Peptide-I; SUP, supernatant.

To increase the clinical relevance of the *in vitro* models, serum was included in the growth media. Again, 10 mM NaSa resulted in decreased growth kinetics (Figure 8A); however, except for 0.1 NaSa, CFU counts were unaffected by NaSa compared to the control (Figure 8B). Ten millimolar NaSa significantly decreased the expression of *agr* in serum-supplemented media after 24 and 48 h (Figure 8C), although *spa*, *hla* and *rnaIII* expression was generally unaffected (Figures 8D–F). Similar to TSB, lower amounts of NaSa (0.1 mM) resulted in an increase in *hla* and *rnaIII* expression (Figures 8E,F), whereas *spa* expression was unaffected by NaSa (Figure 8D).

## Discussion

The *agr* quorum sensing (QS) system is responsible for the direct and indirect regulation of a vast collection of genes in *S. aureus*, many of which are directly involved in virulence (George and Muir, 2007), contributing to the various pathogenic mechanisms observed during *S. aureus* infection. As such, inhibition of *agr* is a potential mechanism for infection control (Tan et al., 2018).

In this study, using the model laboratory strain *S. aureus* ATCC 25923 (*agr* III), we demonstrated that NaSa exhibits a concentration-dependent modulatory effect on *agr* at the gene and protein levels. At higher concentrations (10 mM), sodium salicylate effectively inhibited the expression and transcription of *agrBDCA*. This inhibition caused a decreased downstream secretion of virulence factors under the control of *agr*, such as alpha-toxin, delta-toxin and staphopain. Conversely, at lower concentrations (≤0.1 mM), sodium salicylate significantly increased the expression of *agrBDCA* with secretion levels of virulence factors comparable to those of the TSB control. This dual dose response effect of NaSa (i.e., high concentrations acting as a QS inhibitor vs. low concentrations acting as a QS potentiator) is, to our knowledge, described for the first time; however, the specific mechanism by which sodium salicylate induced modulation of *agr* is still unclear. In another *S. aureus* strain, a similar compound, salicylic acid, has been proposed to interact and bind to four possible sites in AgrA (Dotto et al., 2021), hindering its interactions with target genes, including *agrBDCA*. Furthermore, Queck et al. (2008) demonstrated that the *agr* QS regulon of *S. aureus* is divided into two pathways: (i) RNAIII-independent control of metabolism and PSM cytolysin genes and (ii) RNAIII-dependent control of additional virulence genes. The same study found that PSM expression was regulated by direct binding of AgrA. It could be possible that the regulation of *agrBDCA* and, in particular, the downregulation of *agrA* and the PSM delta-hemolysin could have contributed to the observed effect of the highest dose of NaSa in ATCC 25923. Importantly, the effect of NaSa on regulators of *agr*, including SarA, SarR, and SigB (Lauderdale Katherine et al., 2009; Reyes et al., 2011), and the direct interactions between NaSa and virulence factor genes need to be further elucidated. While the *agr* QS system is responsible for regulating the expression of many virulence factors, it does not possess plenary control. Therefore, it is possible that RNAIII-independent control of metabolism, which is outside the scope of this paper, may produce the repression in virulence factor production. One example of this is catabolite control protein (CcpE), a negative regulator of *hla* (Hartmann et al., 2014) that affects the *in vitro* growth yield of *S. aureus* (Hartmann et al., 2013). In addition, Dotto et al. (2017) showed that salicylic acid treatment induced a switch in the metabolic pathway from the tricarboxylic acid cycle to the fermentative pathway and may modify the expression of catabolite proteins such as CcpE. Due to the observed decrease in growth rate and Hla production here, it needs to be further confirmed if the highest dose of NaSa does affect the metabolism in *S. aureus.*

NaSa also inhibited *agr* QS in another *agr* type strain. A concentration of ≥ 1 mM NaSa significantly downregulated *agrA* and *hld* expression in *S. aureus* 8325-4 wild-type (*agr* I). Using reporter strains of *S. aureus* 8325-4, 10 mM NaSa did not inhibit *rnaIII* expression, while lower concentrations of NaSa (≤ 1 mM) increased the expression of *rnaIII* and upregulated *hla*. This increased expression may be due to the tight regulation of *agr* by other regulators and potentially suggests the influence that NaSa has on these regulators. A recent study has shown that 2 mM salicylic acid exhibits an inhibitory effect on the expression of CodY, a negative regulator of *agr* (Dotto et al., 2017). Thus, it can be speculated that while high concentrations (10 mM) of NaSa inhibit *agr*, at lower concentrations (≤ 1 mM), this inhibition no longer occurs, but repression of CodY remains, resulting in increased expression of the *agr* QS system and some of its products, as observed in this investigation.

*Staphylococcus aureus* produces multiple virulence factors, including alpha hemolysin, delta hemolysin, leukotoxins, proteases, and phenol-soluble modulins (PSMs) (Grumann et al., 2014; Oliveira et al., 2018). Here, it is shown that NaSa modulates the expression of *agr* QS-regulated virulence factors in *S. aureus*. High concentrations of NaSa (10 mM) decreased the expression of delta hemolysin in *S. aureus* both at the RNA and protein levels and decreased the secretion of alpha hemolysin and staphopain A. It is known that the *agr* QS system directly affects RNA III, a regulatory RNA that upregulates delta hemolysin (Novick et al., 1993), alpha hemolysin (Morfeldt et al., 1995; Dunman et al., 2001) and staphopain A (Cheung Gordon et al., 2011). We hypothesize that NaSa inhibits the secretion of delta hemolysin, alpha hemolysin and staphopain A through *agrBDCA-* and *rnaIII-*mediated interactions. It is important to acknowledge that the observation of NaSa on *agr* expression was performed using different analytical techniques to measure the expression of *agrBDCA*, *agrA* and *rnaIII*, which may represent both a limitation because we see strain variations but also a strength because NaSa modulation of *agr* was ascertained in various assays. In future studies, it would be interesting to use the same technique for a more direct comparison between *agrBDCA* and *rnaIII*. Furthermore, this study included only a selection of virulence factors known to be regulated by the *agr* QS system, therefore additional in-depth studies should be carried out including additional virulence factors specific for biofilm formation and toxin production.

RNAIII is also known to downregulate the secretion of *S. aureus* surface proteins, including Protein A (Dunman et al., 2001). Our data showed *spa* expression and secreted protein A levels that are comparable to those of the control in ATCC25923 and 8325-4. Nevertheless, *spa* is not only regulated by *agr* and RNAIII. SarA (Chien and Cheung, 1998; Dunman et al., 2001), SarS (Tegmark et al., 2000), Rot (Saïd-Salim et al., 2003), SarT (Schmidt Katherine et al., 2003) and the ArlR-ArlS two-component system (Fournier et al., 2001) are also involved in the regulation of *spa.* In summary, the observed effect of NaSa (10 mM) as a virulence inhibitor (decreased levels of Hla, Hld and staphopain) in this investigation suggests treatment of *S. aureus* infection with NaSa could result in fewer damaging effects in the host while also limiting immune evasion (no increase in protein A). This could therefore benefit the immune system in clearing an infection, but further studies are needed to assess exactly how NaSa affects the host immune system.

Our group has previously shown that NaSa decreased biofilm formation and the production of virulence factors, including siderophores and pyocyanin, in *Pseudomonas aeruginosa* (Gerner et al., 2020). Furthermore, salicylic acid has been reported to decrease the expression of virulence factors in *S. aureus* (Kupferwasser et al., 2003), *P. aeruginosa* (Sethupathy et al., 2016) and *Escherichia coli* (Pomposiello et al., 2001; Bhaskarla et al., 2016). In general, salicylate may cause environmental xenobiotic stress to opportunistic bacteria,

thus disrupting QS signaling and affecting the downstream expression of virulence factors (Price et al., 2000; Dotto et al., 2017). However, in *E. coli* and *S. aureus*, the relationship between salicylate and the modulation of growth rate, response to antibiotics, and virulence factor expression is double-edged and complex. In *E. coli*, increased resistance to certain antibiotics and upregulation of the antibiotic resistance operon *marRAB* was observed after incubation in salicylate (Cohen et al., 1993). Though this was coupled with an increase in susceptibility to aminoglycosides (Aumercier et al., 1990) and a reduction in virulence factor synthesis (Kunin et al., 1994). A similar observation was made with *S. aureus*, where salicylate treatment increased resistance to fusidic acid (Price et al., 1999) and fluoroquinolones (Gustafson et al., 1999) while also increasing susceptibility to vancomycin (Nicolau et al., 1995), and, as shown in this study, an increase in susceptibility to rifampicin. However, comparison of antimicrobial susceptibility data between studies should be done with caution, as experimental conditions such as dosage, strain and culture media can influence the result.

Biofilm formation is a key adaptation that allows *S. aureus* to establish chronic infection. Contrary to their planktonic counterparts, biofilm bacteria exhibit a modified phenotype regarding growth kinetics, protein production and gene expression, providing the characteristic recalcitrance to antibiotics (Lewis, 2010) and the ability to resist the host immune response (Lewis, 2005). The role that *agr* plays in biofilm maintenance is well-characterized (Kong et al., 2006; Boles and Horswill, 2008; Beenken et al., 2010). Several clinical studies have shown that dysfunction in the *agr* operon results in greater pathogenicity, harder to clear infection and typically impaired patient outcome and morbidity (Schweizer et al., 2011; Derakhshan et al., 2021). Various studies into QS inhibitors (QSIs) have correlated comparable information regarding biofilm formation in *S. aureus*, both *in vitro* and *in vivo.* For example, working with a similar compound, salicylic acid, Dotto et al. (2021) found that it stabilizes biofilm formation in *S. aureus* by reducing the production of PSMs, a family of small proteins involved in instigating biofilm dispersal due to their detergent-like properties (Dotto et al., 2021). A similar result was observed by Mahdally et al. (2021) in an investigation of Staquorsin, another QSI, in which treated *S. aureus* produced thicker biofilm, equivalent to that of an *agr-* mutant (Mahdally et al., 2021). In this study, the effect of NaSa on biofilm formation in the *S. aureus* lab strain ATCC 25923 as well as in clinical isolates obtained from PJI and wound infection was investigated. The growth of *S. aureus* ATCC 25923 in NaSa produced phenotypic effects that correspond with the published literature, resulting in an increase in biofilm biomass on polystyrene and on titanium surfaces at 10 mM NaSa. Because 10 mM NaSa inhibited *agrBDCA* expression and Hld secretion, to further investigate whether the increase in biofilm biomass of ATCC 25923 on polystyrene was the result of a decrease in dispersal events, viable planktonic cells released from the biofilm to the surrounding medium and viable cells inside the biofilm were quantified at 24 and 48 h. Significantly fewer CFU were identified in the planktonic phase after treatment with 10 mM NaSa, with no significant change in total CFU in the biofilm after 48 h. This suggests that NaSa influences phenotypic biofilm formation, which is explained by the observed downregulation of *agr* followed by downregulation of the PSM delta-hemolysin resulting in a decrease in biofilm dispersal and consequently greater biofilm formation on PS and Ti surfaces under static conditions in ATCC 25923. However, in planktonic experiments, NaSa (10 mM) treatment was also shown to reduce the growth rate of *S. aureus.* As such, it is possible that the reduction in viable colonies observed may be due to both growth inhibition and inhibition of biofilm dispersal.

For clinical relevance, biofilm formation was also investigated in clinical strains isolated from PJI on titanium surfaces and from wound infections on collagen-coated surfaces. We also aimed to characterize whether this biofilm response was contiguous across all four known *agr* types. Biofilm formation across each of the surface types, isolate origins, and *agr* types under study did not show a consistent response, although a general trend of increased biofilm formation after NaSa treatment with the highest concentration was observed. The heterogeneity in biofilm expression across the four *agr* types in the different experimental models suggests varying levels of *agr* modulation at the phenotypic level. Because AgrA is conserved across all *agr* types (Tan et al., 2018), in theory it is expected, based on the experiments performed by Dotto et al. (2021) to observe a similar phenotypic response across each *agr* type if the effect of sodium salicylate is brought about through interactions with AgrA as it is with salicylic acid. As such, there is potential for other mechanisms to be involved in the interactions between NaSa and *agr*. As previously described, there is a significant level of interplay and cross-regulation in *S. aureus* regulatory operons such as *SarA* and *SigB*. The formation, maintenance and dispersal of biofilms are incredibly complex and regulated by these operons. Therefore, a simple one-to-one treatment-response relationship may be naïve to assume.

The potential application for QS inhibitors lies not only in the modulation or inhibition of bacterial communication but also in their application alongside traditional treatment methods to potentiate their efficacy. The use of QS inhibitors to potentiate the effect of antimicrobial agents has been previously demonstrated, including sodium salicylate in combination with silver (Gerner et al., 2021) and in combination with antibiotics (Polonio Roy et al., 2001; Brackman et al., 2011; Belfield et al., 2017), showing increased MBEC susceptibility of *S. epidermidis* to vancomycin (Polonio Roy et al., 2001). In this study, exposure to 10 mM NaSa resulted in a twofold decrease in the MBEC of rifampicin (from 1,024 to 512 μg/mL). This may be due to the less viable *S. aureus* recovered from the biofilm before treatment, therefore reducing the load under challenge. This ability to potentiate the effect of antibiotics that possess unrelated mechanisms of action and that work against both Gram-positive and Gram-negative species suggests a broader spectrum application as a cotreatment to traditional antibiotic therapy. On the other hand, culture of *S. aureus* in an environment containing salicylate also presents the risk of developing resistance against other antimicrobial agents (Price et al., 1999; Riordan et al., 2007). This has been suggested to be a result of the reduction in growth rate exhibited at higher salicylate concentrations but also as a result of the molecular and metabolic variations induced (Riordan et al., 2007; Dotto et al., 2017). Moreover, there is a concern that inhibitory effects on *agr* can increase biofilm tolerance due to increased biofilm formation *via* reduced dispersal (Dotto et al., 2021), although this is not always the case (Yarwood et al., 2004) and is most likely substance-, strain- and assay dependent, as suggested by this investigation. Even though NaSa increased *S. aureus* ATCC 25923 biofilm formation on both titanium and polystyrene, there was no increased tolerance against silver, a common antiseptic used in wound care, or against a wide range of different antibiotics.

To increase the clinical relevance of the *in vitro* systems, serum was included in the media. Although serum proteins can bind to and decrease the potency of many compounds (Trainor, 2007), a concentration of 10 mM NaSa still inhibited *agr* activity. However, *hla* expression was not affected by NaSa in the presence of serum after 24 h. In the media control sample, serum resulted in increased *rnaIII* and *hla* expression compared to TSB without serum (data not shown). Both calf serum (Oogai et al., 2011) and human serum (Malachowa et al., 2011) have previously been shown to increase *hla* expression in *S. aureus* when compared to traditional TSB cultures, highlighting the importance of serum supplementation when appropriate.

The observed biphasic concentration-dependent effects, where high and low concentrations of NaSa decreased and increased, respectively, and the QS activity and expression of certain virulence factors could have important implications for potential future clinical applications. Factors such as local NaSa tissue concentration, bioavailability and pharmacokinetics should be carefully investigated alongside the host response *in vivo*.

## Conclusion

Sodium salicylate functioned as a quorum sensing inhibitor in *S. aureus* in the presence and absence of serum, decreasing toxin production levels with some modulation of biofilm formation. The observed effect on biofilm formation seemed to depend on the characteristics of the strain and material surface utilized. These results demonstrate the *in vitro* QS-inhibitory and anti-virulence effects of NaSa against *S. aureus*. This should be further evaluated *in vivo* to confirm whether interfering with bacterial communication is a potential alternative or adjuvant to traditional antibiotics/antiseptics for infection control of *S. aureus* causing wound- and periprosthetic joint infections.

## Data availability statement

The original contributions presented in this study are included in the article/Supplementary material, further inquiries can be directed to the corresponding author/s.

## Author contributions

MW, PT, SA, and MT contributed to the conception and critically revised the manuscript. AT, EG, RF, ME, and MT contributed to the design of the study, performed the experimental work and data analysis, interpreted the data, and drafted the manuscript. All authors contributed to manuscript revision and approved the submitted version.
