# Supplementary figures and images for: Role of sodium salicylate in Staphylococcus aureus quorum sensing, virulence, biofilm formation and antimicrobial susceptibility
Source: Front Microbiol. 2022 Aug 1;13:931839. doi: 10.3389/fmicb.2022.931839 (PMC9384861; doi:10.3389/fmicb.2022.931839)

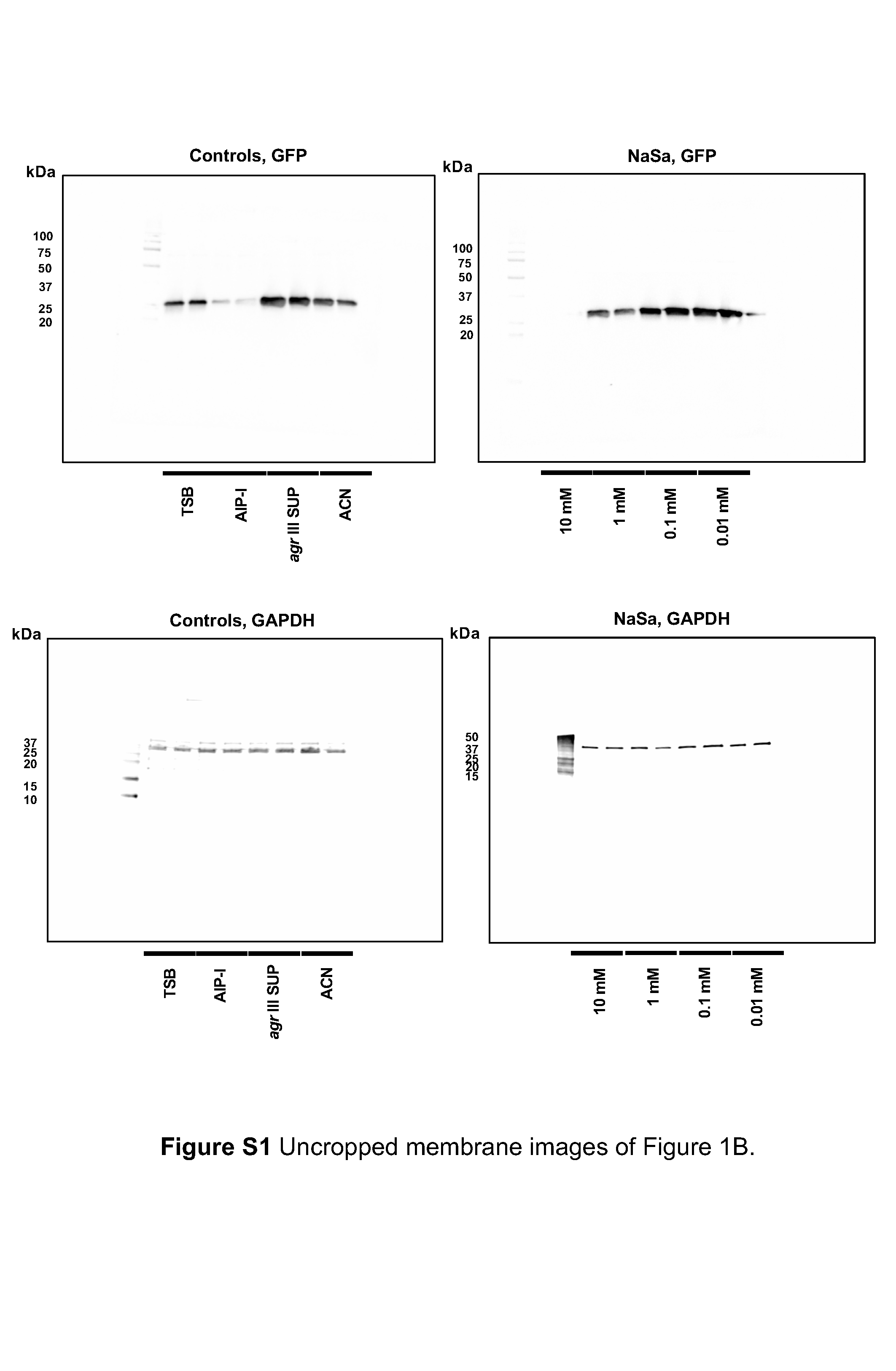

Supplement: Supplementary file 1 [file Image_1.tiff]

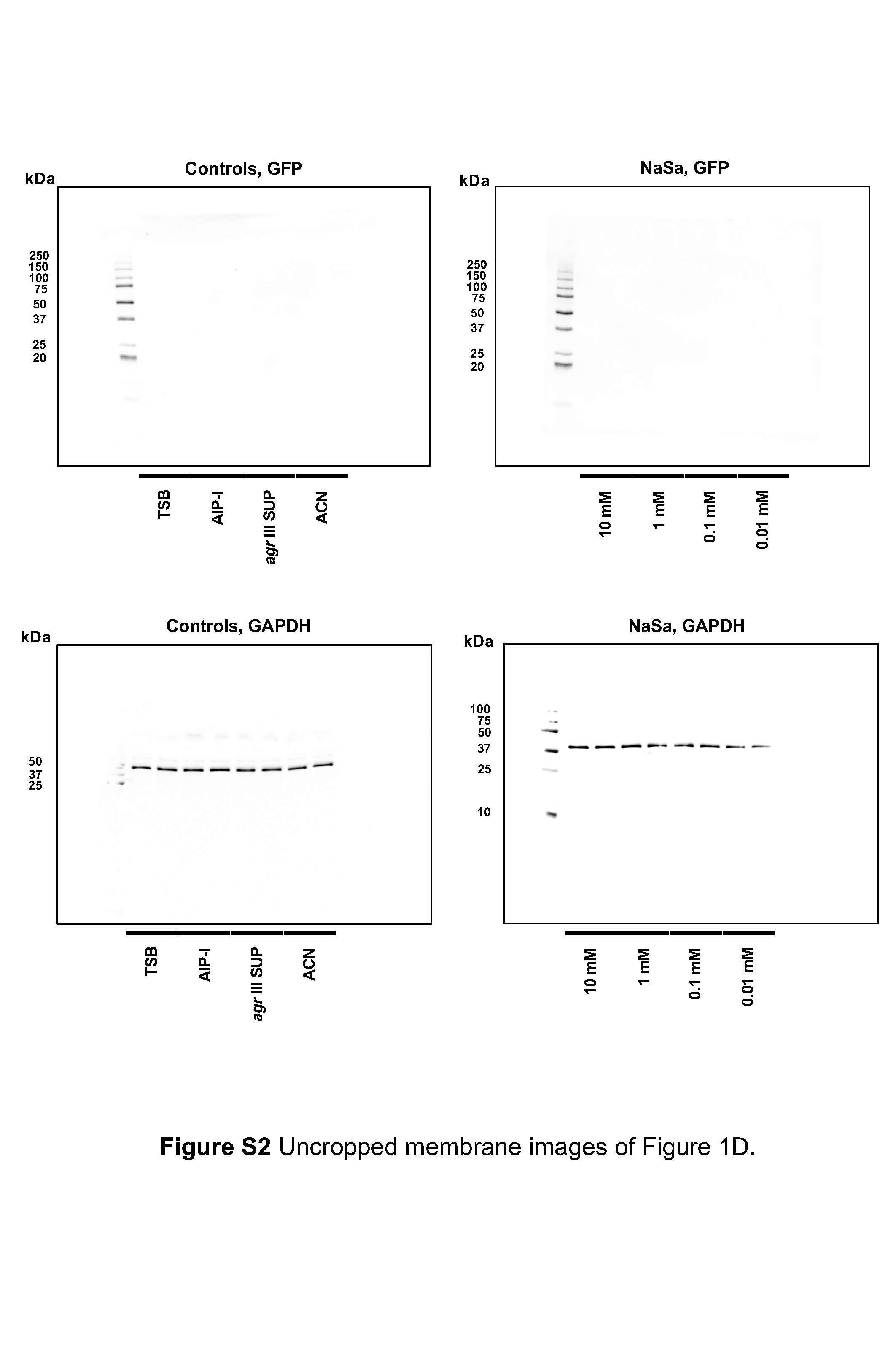

Supplement: Supplementary file 2 [file Image_2.tiff]

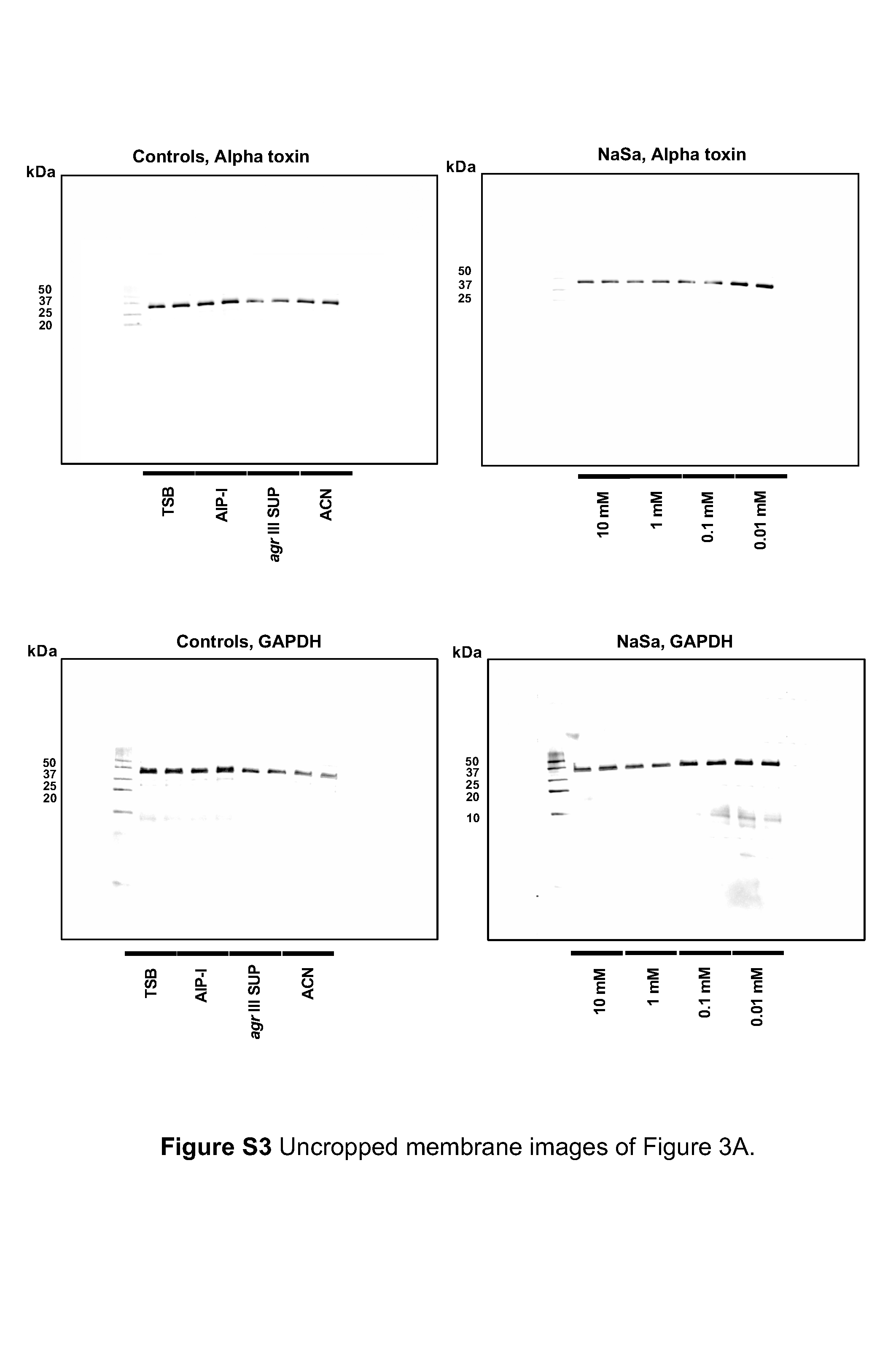

Supplement: Supplementary file 3 [file Image_3.tiff]

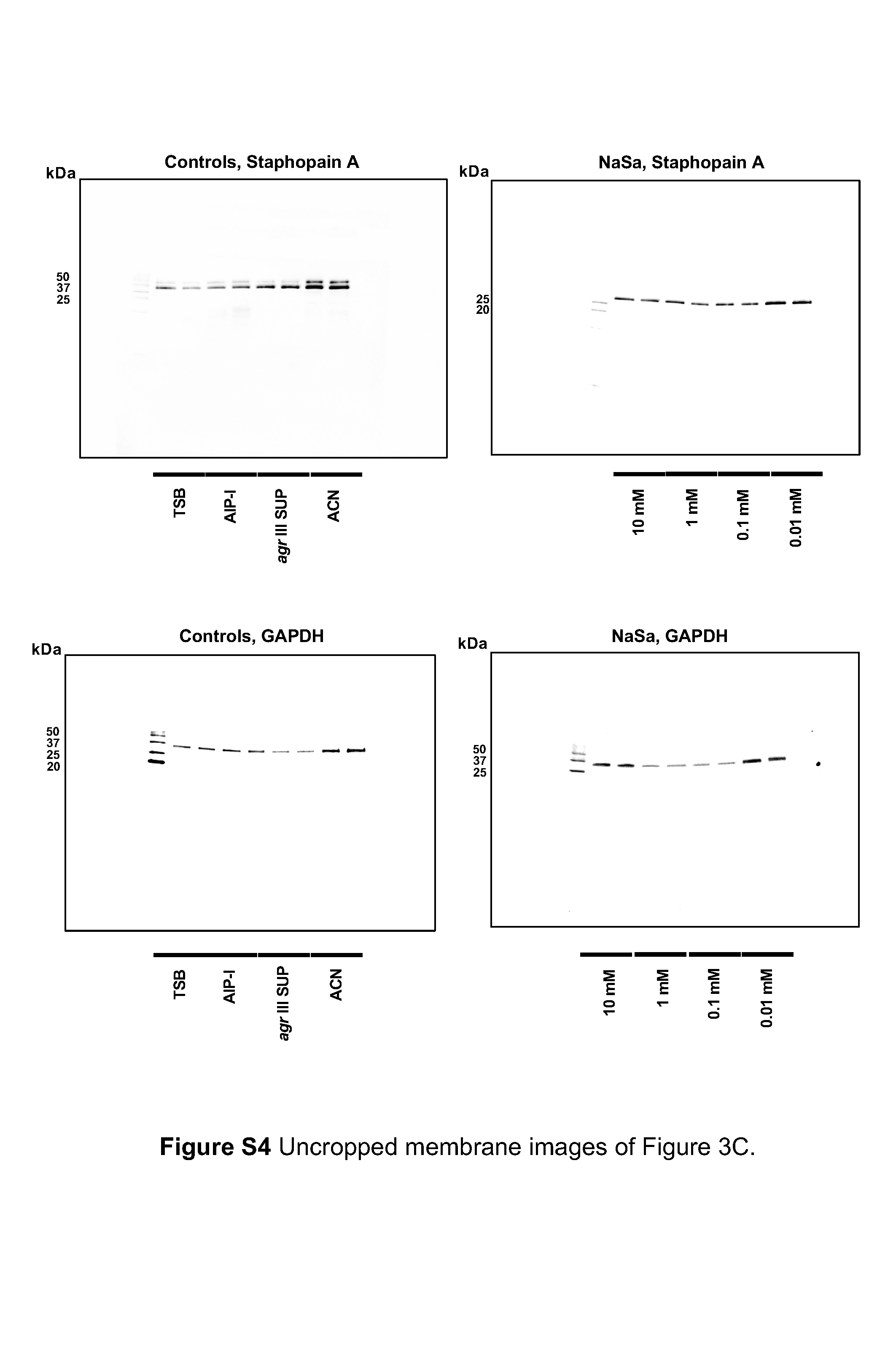

Supplement: Supplementary file 4 [file Image_4.tiff]

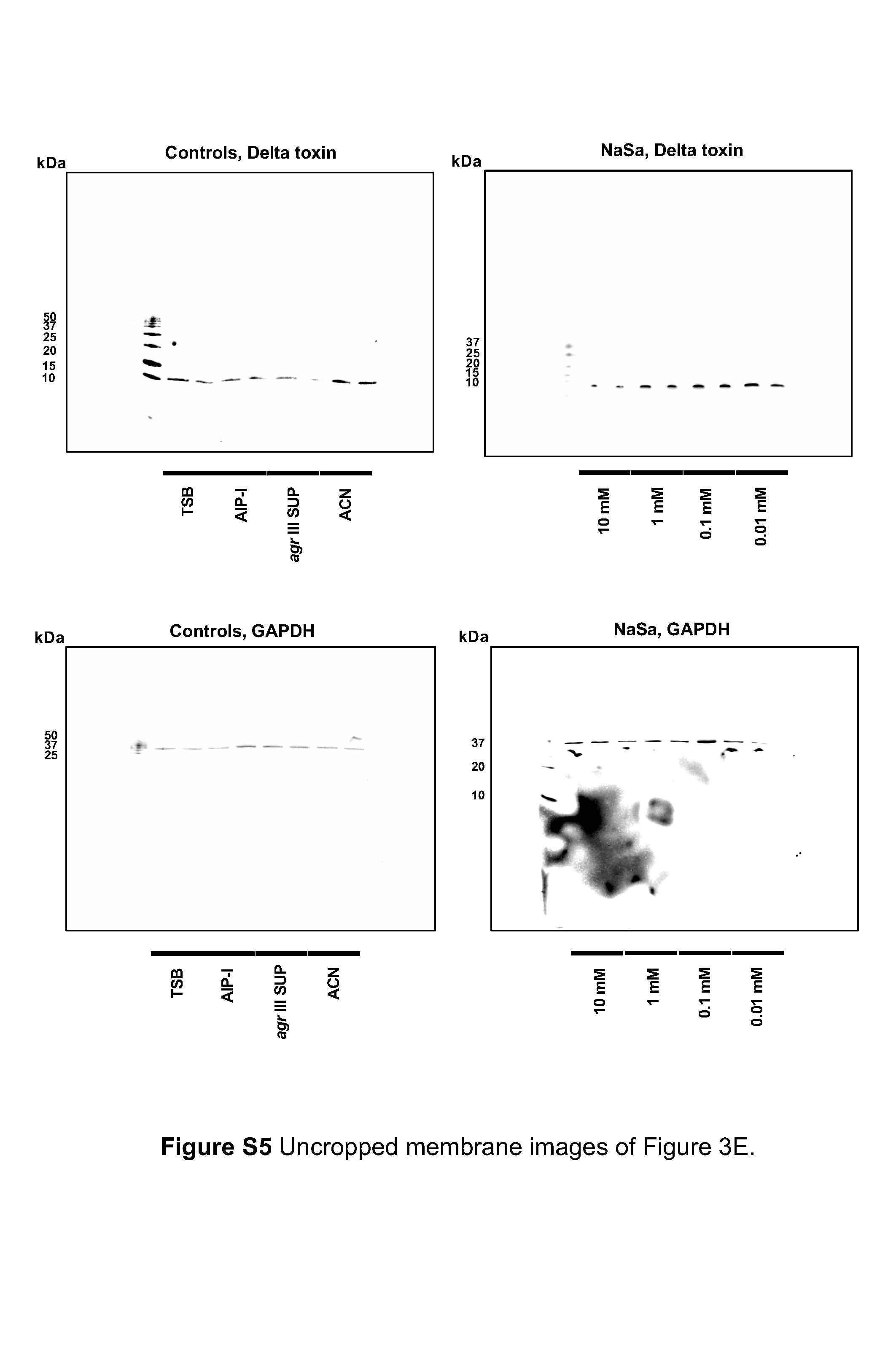

Supplement: Supplementary file 5 [file Image_5.tiff]

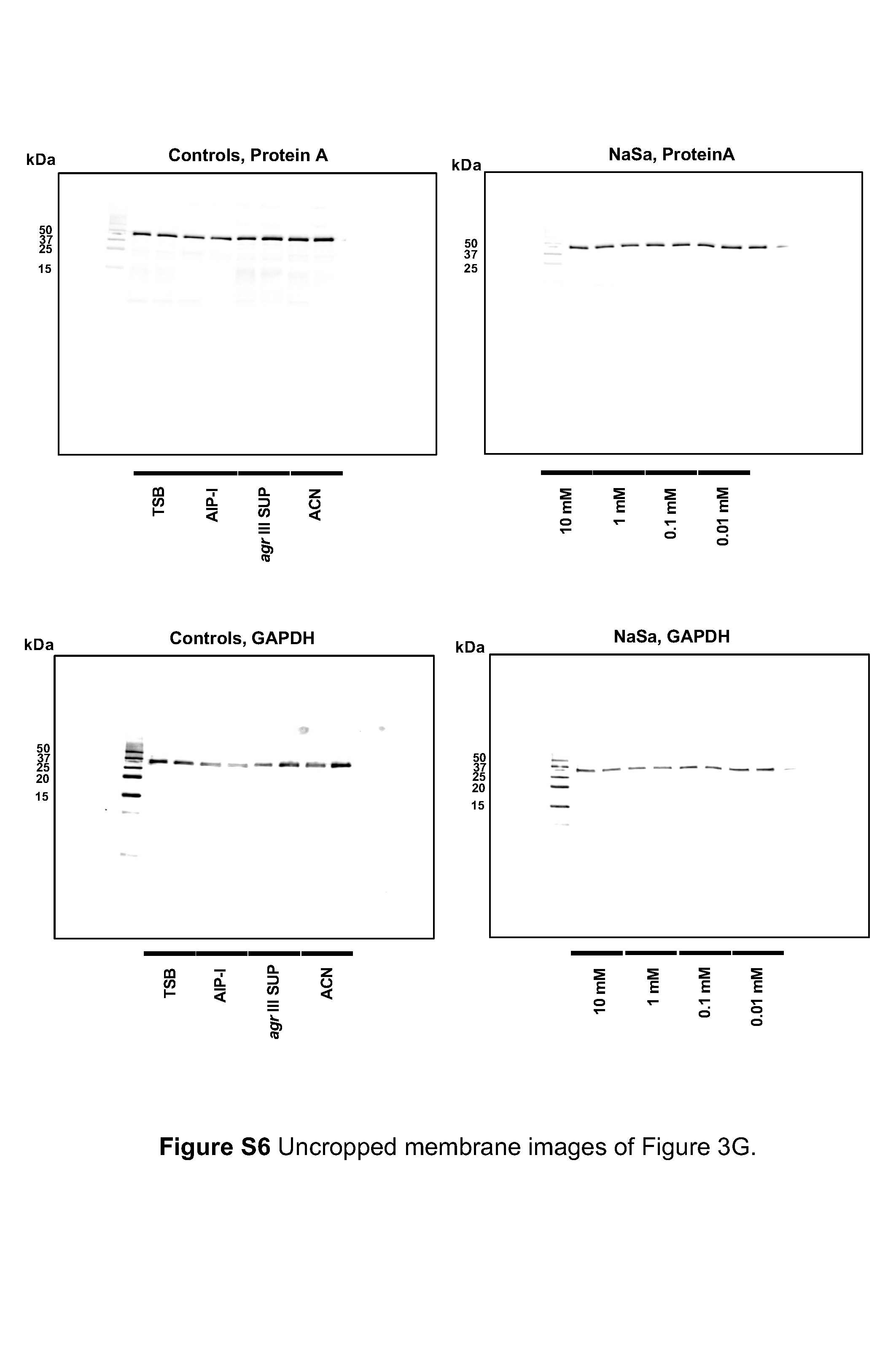

Supplement: Supplementary file 6 [file Image_6.tiff]

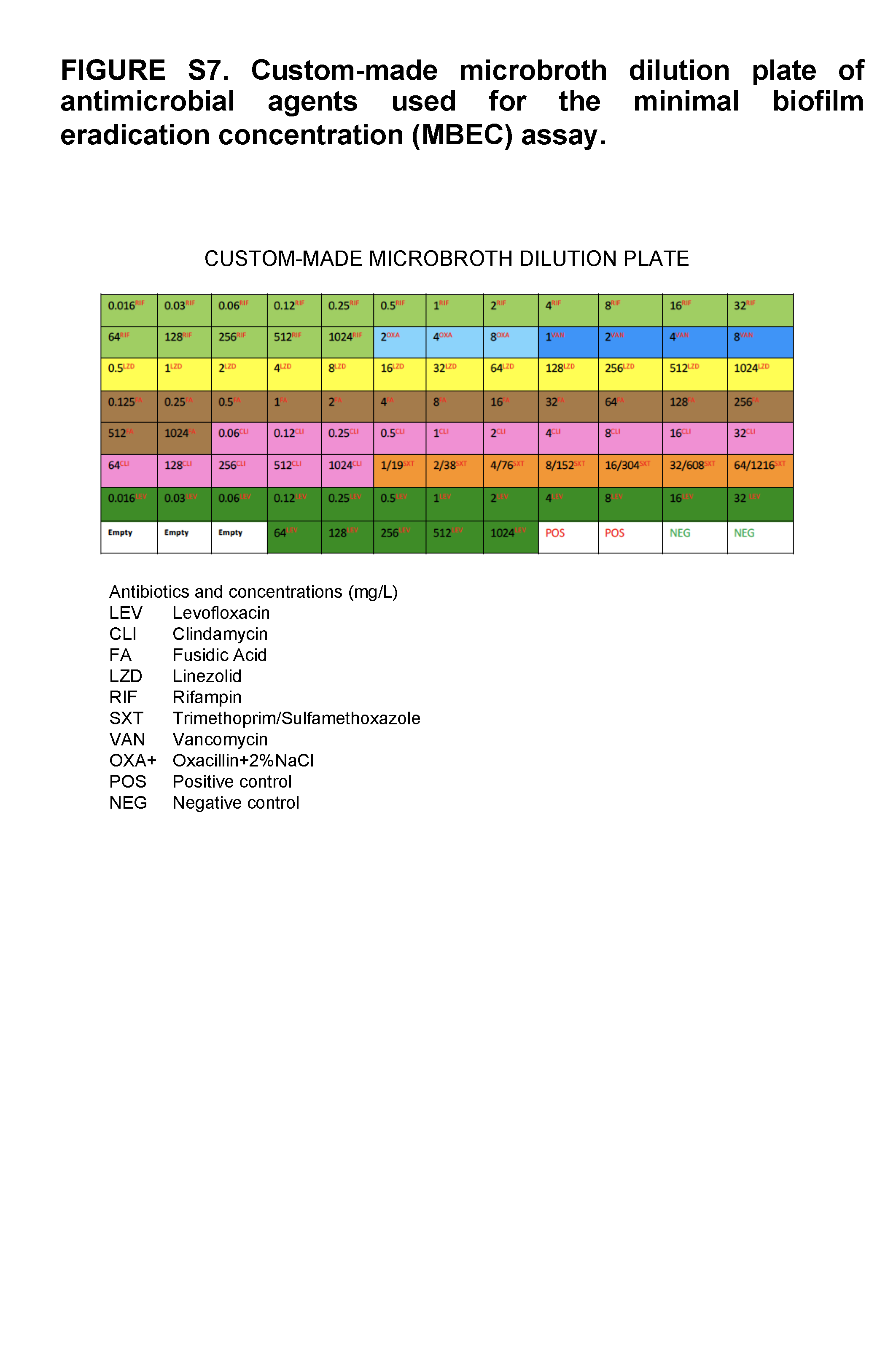

Supplement: Supplementary file 7 [file Image_7.tiff]
